# Microbiota‐gut‐brain axis multi‐organ chip construction and applications in drug evaluation

**DOI:** 10.1002/imo2.70065

**Published:** 2025-11-24

**Authors:** Yue Tang, Hewen Chen, Ziyue Zhao, Xuesong Kang, Wenxin Wang, Kun Dai, Yufei Guo, Axin Liang, Aiqin Luo, Zikai Hao

**Affiliations:** ^1^ Key Laboratory of Molecular Medicine and Biotherapy, the Ministry of Industry and Information Technology, School of Life Science Beijing Institute of Technology Beijing China; ^2^ Advanced Technology Research Institute Beijing Institute of Technology Jinan China; ^3^ Institute of Environmental Biology and Life Support Technology, School of Biological Science and Medical Engineering Beihang University Beijing China; ^4^ Shandong Institute for Food and Drug Control Jinan China; ^5^ Department of Statistics the George Washington University Washington District of Columbia USA

**Keywords:** drug evaluation, microbiota‐gut‐brain axis, microfluidics, organ‐on‐a‐chip

## Abstract

The theoretical framework of the microbiota‐gut‐brain axis (MGBA) elucidates the influence of gut microbiota (GM) on the central nervous system (CNS) and offers novel approaches for diagnosing and treating neurological disorders. The application of microfluidic organ chips to study this axis represents an innovative methodology, enabling multidisciplinary assessment of drug effects. Bionic microphysiological systems based on these chips establish a groundbreaking paradigm for analyzing cross‐organ interaction mechanisms within the MGBA. This technology facilitates systematic evaluation of pharmacokinetics, host‐microbe coevolution, and neuroendocrine regulation, thereby supporting multimodal cross‐validation in pharmacodynamics, toxicology, and translational medicine. This review summarizes the conceptual foundation and mechanistic insights of the MGBA, highlights recent advances in its constituent Multiorgan components, including gut‐on‐a‐chip, blood‐brain barrier‐on‐a‐chip, and brain‐on‐a‐chip models, alongside multiorgan chip cascade technologies, and underscores their applications in drug evaluation. Additionally, it discusses future challenges and developmental directions for the MGBA multiorgan chip technologies in this field.

## Highlights

- Systematically integrating the microbiota‐gut‐brain axis (MGBA) with multiorgan chip technology to construct a research paradigm for elucidating the mechanisms of cross‐organ interactions.
- Providing a comprehensive elaboration on the technical iterations and current development status of core models, including gut‐on‐a‐chip, blood‐brain barrier‐on‐a‐chip, brain‐on‐a‐chip, as well as multiorgan cascading techniques.
- Discussing the critical application value of MGBA organ‐on‐a‐chip platforms in drug screening and preclinical drug research, including pharmacokinetic studies, pharmacodynamic profiling, and toxicity evaluation.

## Graphical abstract

Microbiota‐gut‐brain axis multiorgan chip and its applications in drug evaluation. The chip includes essential biological components such as gut microbiota, gut barrier, blood‐brain barrier (BBB), and brain. This integrated platform incorporates neural (blue), endocrine (yellow), immune (pink), and metabolic (green) pathways to investigate the impact of drugs on neural function, BBB permeability, microbiota–host interactions, and gut barrier function. Furthermore, the platform is utilized for mechanism analysis of cross‐organ interactions, drug screening, and preclinical drug research, such as pharmacokinetics, pharmacodynamics, and toxicity evaluation. It also supports the development of microbiota modulation strategies and provides innovative tools for targeted drug delivery for neurological diseases.

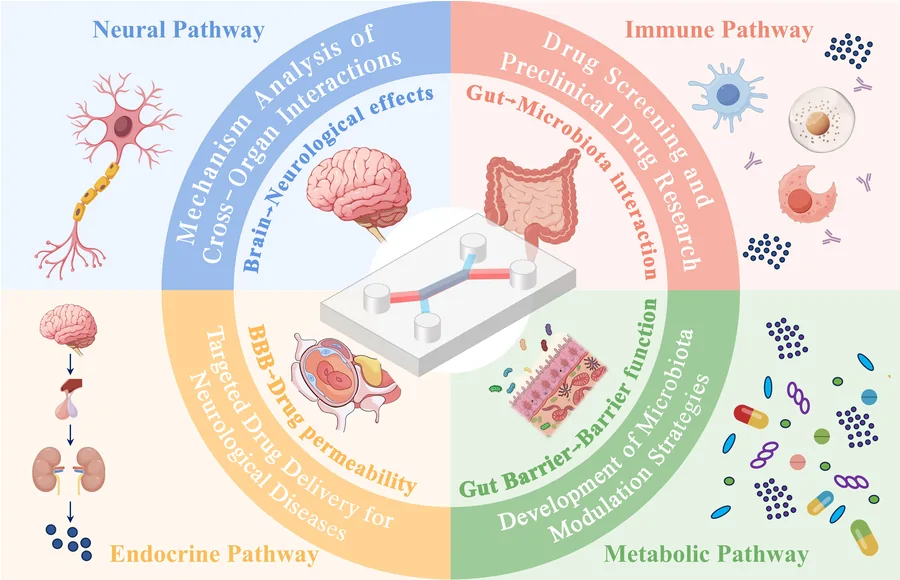

## 1 INTRODUCTION

The human gut harbors a vast microbial community, numbering in the trillions, which forms a symbiotic relationship with the host and deeply engages in physiological processes such as metabolism and immunity [1]. The stability of its composition and function directly impacts human health [2], with dysbiosis being significantly associated with metabolic and immune disorders, including inflammatory bowel diseases (IBDs) [3], type 2 diabetes [4], cardiovascular diseases [5], and irritable bowel syndrome (IBS) [6]. More notably, disruptions in the gut microbiota (GM) can contribute to neurocognitive dysfunction through the microbiota‐gut‐brain axis (MGBA), establishing causal links with neurological conditions such as depression [7], anxiety [8], schizophrenia [9], and dementia [10, 11]. The MGBA, a bidirectional regulatory system integrating neural, endocrine, immune, and metabolic pathways between the gut and brain [12], offers a novel microbiota‐based approach for treating neurological disorders. However, traditional animal models and in vitro culture systems face limitations in replicating the complex human physiological environment and dynamic organ interactions due to ethical constraints and experimental conditions, thereby compromising the accuracy and reliability of research outcomes [13].

The organ‐on‐a‐chip (OoC) is a bionic system engineered using microfluidic technology to simulate the microenvironmental characteristics of human organs in vivo [14]. Compared to traditional animal experiments and cell cultures, this technology offers advantages such as low sample consumption, high throughput, and precise microenvironment control, enabling the construction of multiorgan chip (MOC) systems for comprehensive pharmacodynamic evaluation of drug mechanisms. OoCs demonstrate significant technical advantages in drug absorption, metabolism, and toxicity studies [15], providing a critical platform for drug screening and development. By leveraging microfluidic technology to model the MGBA in vitro, researchers can investigate the therapeutic effects of GM on CNS disorders in physiologically relevant conditions, thereby advancing innovative drug discovery.

Current studies often focus on a single dimension of OoC technology, either emphasizing the correlation between GM and diseases [16] or analyzing OoC techniques in isolation [17]. This article pioneers the systematic integration of two cutting‐edge fields: the MGBA and MOC technology. By breaking down the silos between descriptive correlations and technological principles. The article not only comprehensively reviews traditional methods for constructing OoC systems, but also critically examines innovative designs for gut‐blood‐brain barrier (BBB)‐brain cascade MGBA chips. It highlights their indispensable role in mechanistic studies, such as the trans‐organ transport of microbiota metabolites and real‐time monitoring of neuroimmune interactions, while also exploring specific pathways for applying MGBA‐MOC technology in neurological drug screening. It is important to note that the literature selection for this review centers on the core direction of how OoC systems evolve from laboratory models to tools for solving clinical problems. The review focuses specifically on the construction and functional expansion of OoC platforms. Temporally, it traces the iterative process of OoC technologies—from initial physiological simulation, to reconstitution of complex physiological processes, and finally to clinical translation. Simultaneously, priority is given to studies published in high‐impact journals and work from core research groups in the field. This approach ensures that classic or pioneering studies are introduced and discussed at each stage, resulting in a literature corpus that is both cutting‐edge and systematically structured. In conclusion, this article centers on MGBA‐MOC technology, providing a systematic review of its underlying mechanisms, technological advancements, and applications in drug screening. Despite being in the developmental stage, the unique advantages of this technology suggest promising prospects for future disease treatment research.

## 2 OVERVIEW OF MICROBIOTA‐GUT‐BRAIN AXIS MECHANISMS

**Figure 1:**
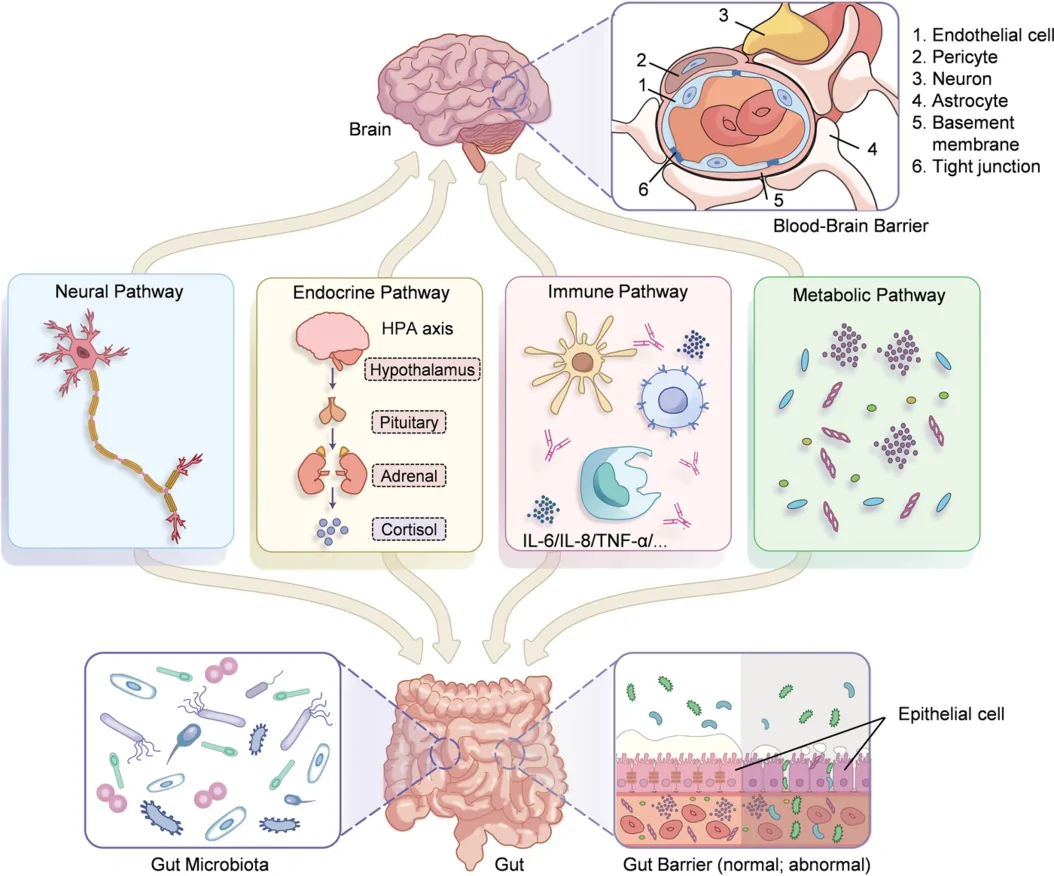
The communication pathways of the microbiota‐gut‐brain axis (MGBA). The MGBA transmits bidirectional signals between the gut and the brain through neural (blue), endocrine (yellow), immune (pink), and metabolic (green) signaling pathways. Substances derived from the neuroendocrine‐hypothalamic‐pituitary‐adrenal (HPA) axis, enteric immune system, and gut microbiota‐derived metabolites can mediate the gut microbiota's effects on the brain only after traversing the gut barrier to enter systemic circulation and crossing the blood‐brain barrier.

The communication mechanisms of the MGBA rely on the synergistic interactions among the nervous, endocrine, immune systems, and microbial metabolites (Figure 1). Within this intricate network, the vagus nerve's afferent and efferent fibers are intertwined, forming the most direct neural pathway linking the gut and the brain. Vagal afferents transmit chemical signals as crucial gut inputs; neurohormones secreted by enteroendocrine cells, such as cholecystokinin, glucagon‐like peptide‐1, and 5‐hydroxytryptamine, specifically bind to chemosensors on vagal afferent terminals, including cholecystokinin a receptor, glucagon‐like peptide‐1 receptor, and 5‐hydroxytryptamine 3 receptor [18]. Additionally, microbial metabolites produced by GM, such as γ‐aminobutyric acid (GABA) and bacterial flagellin, bind to GABA‐B and TLR5 receptors, respectively, triggering action potentials [19, 20]. These signals are transmitted via afferent fibers to the central nervous system's (CNS's) dorsal motor nucleus of the vagus and area postrema, subsequently projecting to brain regions like the hypothalamus and amygdala to regulate appetite, mood, and stress responses [21, 22]. Vagal efferent fibers, through preganglionic parasympathetic fibers, control target organs such as the gut, heart, and lungs, modulating intestinal motility, secretion, and local immunity [23, 24]. Animal studies and clinical findings indicate that vagotomy can significantly impact brain function and alter host behavior [25]. In mouse models, post‐antibiotic intervention, subdiaphragmatic vagotomy has been shown to reverse depressive‐like behaviors and anhedonia phenotypes induced by *Lactobacillus intestinalis* and *Lactobacillus reuteri* [26]. Early research has also established a correlation between impaired vagal function and increased incidence of psychiatric disorders. Vagus nerve stimulation (VNS) is widely recognized as a potential therapeutic approach for various mental health conditions, including depression, panic disorders, and schizophrenia [27, 28].

The hypothalamic‐pituitary‐adrenal (HPA) axis serves as a central neuroendocrine pathway in gut–brain interactions, with its homeostasis maintained through the cascade release and negative feedback regulation of several key modulatory molecules [29]. Neuroendocrine neurons in the hypothalamus secrete corticotropin‐releasing hormone, which stimulates the pituitary gland to release adrenocorticotropic hormone. Upon entering the circulation, adrenocorticotropic hormone induces the adrenal glands to synthesize cortisol and other glucocorticoids. Cortisol is not only a critical molecule in regulating neurodevelopment and cognitive function but also mediates bidirectional gut–brain communication through multiple pathways. Its receptors are widely expressed in intestinal epithelial cells, immune cells, and enteroendocrine cells [30, 31], directly altering intestinal transit time, permeability, and nutrient availability, thereby reshaping the composition and metabolic activity of the GM [32, 33]. Additionally, cortisol binds to glucocorticoid receptors in brain regions such as the hippocampus, amygdala, and prefrontal cortex, modulating central stress circuits [34].

The dynamic interplay between the HPA axis and the microbiota constitutes another crucial dimension. Gut microbiota can indirectly activate central stress circuits via vagal and enteric nervous system sensory neurons [35]. Animal experiments have confirmed the reciprocal regulatory effects of microbiota on the HPA axis—compared to specific pathogen‐free rats, germ‐free rats exhibit more pronounced neuroendocrine responses and evident anxiety‐like behaviors under stress [36], directly demonstrating the “buffering effect” of GM on the HPA axis's negative feedback homeostasis. When the body is subjected to environmental stimuli, the stress response of the HPA axis further highlights its pivotal role in the gut–brain axis: increased corticotropin‐releasing hormone secretion under stress → adrenocorticotropic hormone drives cortisol synthesis → cortisol alters the intestinal microenvironment while also regulating central functions through the BBB or neural pathways; the activation of hypothalamic glucocorticoid receptors by cortisol ultimately forms a negative feedback loop to terminate the stress response. This dynamic network of stress triggering‐microbiota buffering‐negative feedback termination profoundly elucidates the central role of the HPA axis in maintaining the homeostasis of the gut‐brain system.

The gastrointestinal tract, being the most densely populated region of immune cells in the human body, hosts trillions of microorganisms that drive multi‐tiered immune activities—including central immune remodeling, immune cell migration, and bidirectional neuro‐immune interactions—through their metabolites and pathogen‐associated molecular patterns, profoundly mediating gut‐brain signaling.

When microbial imbalance leads to bacterial death, the released lipopolysaccharide (LPS) can disrupt the intestinal epithelial barrier, increasing gut permeability. LPS activates the TLR4 receptor on macrophages, triggering the NF‐κB signaling pathway, which promotes the release of pro‐inflammatory cytokines such as IL‐6 and TNF‐α. These cytokines, via the bloodstream, breach the BBB, activating TLR2/4 receptors on brain endothelial cells, inducing NLRP3 inflammasome assembly, and polarizing microglia from the anti‐inflammatory M2 type to the pro‐inflammatory M1 type, thereby initiating microglial activation and neuronal damage [37, 38]. The microbiota also regulates the migration of immune cells to the CNS. Intestinal dendritic cells, under microbial stimulation, express the CCR7 receptor and migrate along the CCL19/21 gradient to mesenteric lymph nodes, activating T cell subsets. These T cells enter the circulation through the lymphatic system and some home to the CNS [39]. Additionally, vagal sensory neurons express TLR4 receptors and can directly respond to intestinal LPS signals. Activated vagal signals are transmitted via the nucleus tractus solitarius to the paraventricular nucleus of the hypothalamus, inhibiting HPA axis activation and reducing peripheral corticotropin‐releasing hormone release, thus modulating systemic immune responses [40].

GM, as a critical component of the MGBA, utilizes its metabolites as essential mediators for gut‐brain communication. These metabolites can act through the bloodstream, lymphatic system, or directly on vagal and spinal afferent neurons, participating in core pathways such as direct neurotransmitter regulation, immune signaling mediation, and BBB remodeling [41, 42].

Neurotransmitters synthesized by GM can directly activate receptors in the CNS, establishing a direct signaling pathway from the gut‐vagus nerve‐brain regions. For instance, GABA produced by *Lactobacillus* species specifically binds to GABA‐B receptors on vagal afferent fibers. This signal is transmitted via the nucleus tractus solitarius in the brainstem to limbic system regions like the amygdala and hippocampus, significantly reducing anxiety‐like behaviors [43]. Microbial metabolites can also target receptors on central immune cells, directly regulating neuroinflammatory activities and mediating cross‐system interactions between gut immunity and central inflammation. Indole‐3‐propionic acid, produced by *Clostridium* species, when binding to the aryl hydrocarbon receptor on microglia, inhibits NLRP3 inflammasome assembly, thereby reducing IL‐1β release [44]. Intervention with *Faecalibacterium prausnitzii* increases short‐chain fatty acid levels in the rat cecum and plasma IL‐10 concentrations while inhibiting the release of stress‐related factors such as corticosterone, C‐reactive protein, and IL‐6 [45]. The important immunomodulatory substance butyrate it produces, binds to GPR109A receptors on microglia, inhibiting the NF‐κB pathway and promoting their conversion to the anti‐inflammatory M2 type. Butyrate also inhibits histone deacetylase, upregulates brain‐derived neurotrophic factor (BDNF) expression, enhances the anti‐inflammatory phenotype of microglia, and reduces central inflammation [46]. Moreover, microbial metabolites can regulate BBB integrity, determining the efficiency of peripheral signal transmission to the CNS, acting as a barrier valve in gut‐brain communication. Trimethylamine N‐oxide, produced by *Escherichia* species, when binding to receptors on cerebral vascular endothelial cells, enhances the expression of tight junction proteins occludin and claudin‐5, reducing LPS transport across the BBB [47].

The MGBA achieves dynamic interactions between gut microecology and central functions through a four‐dimensional synergy of neural sensing‐endocrine cascade‐immune remodeling‐metabolite mediation. In this framework, the vagus nerve provides a rapid neural pathway for immediate signal transmission; the HPA axis constructs a slower endocrine feedback loop for sustained regulation; the immune system mediates cross‐organ inflammatory communication, linking peripheral and central immune responses; microbial metabolites serve as signaling molecules that precisely regulate neural, immune, and barrier functions. Both animal models and clinical evidence have confirmed the central role of the MGBA in maintaining brain homeostasis and mediating the onset of psychiatric disorders. This understanding has also provided theoretical targets for microbiome‐based treatments of neuropsychiatric diseases.

## 3 MICROBIOTA‐GUT‐BRAIN AXIS‐RELATED MICROFLUIDIC ORGAN‐ON‐A‐CHIP CONSTRUCTION AND APPLICATIONS IN DRUG EVALUATION

The MGBA multiorgan interaction model, engineered using microfluidic chip technology, has achieved significant advancements under laboratory conditions. Researchers have successfully established precise gut‐, BBB‐, and brain‐on‐a‐chip systems, enabling systematic quantification of drug trans‐barrier transport, neuroactive metabolite dynamics, and host–microbe interactions. This platform provides critical insights into the pathogenesis of neurodegenerative and psychiatric disorders and accelerates the development of innovative therapeutics.

### 3.1 Gut‐on‐a‐Chip

The intestine, a central organ for digestion and absorption in the human body, exhibits distinct roles across its different segments in terms of physiological function, microbial community structure, and drug metabolism. The small intestine is primarily focused on efficient digestion, absorption, and rapid substance transport, with its villi and microvilli structures significantly expanding the absorptive surface area. In contrast, the large intestine emphasizes water reabsorption, fermentation of undigested materials, and maintaining a stable anaerobic microbial community. Current research often lumps both together under the general term intestine, overlooking the divergent functional needs of each segment in chip design. Indeed, precisely simulating the physiological microenvironments of different intestinal segments is a fundamental starting point for the development of gut‐on‐a‐chip (GoC) technology—small intestine chips need to concentrate on dynamic mechanical stimuli and rapid substance transport, while large intestine chips require adaptation to three‐dimensional structures and stable anaerobic environments.

**Figure 2:**
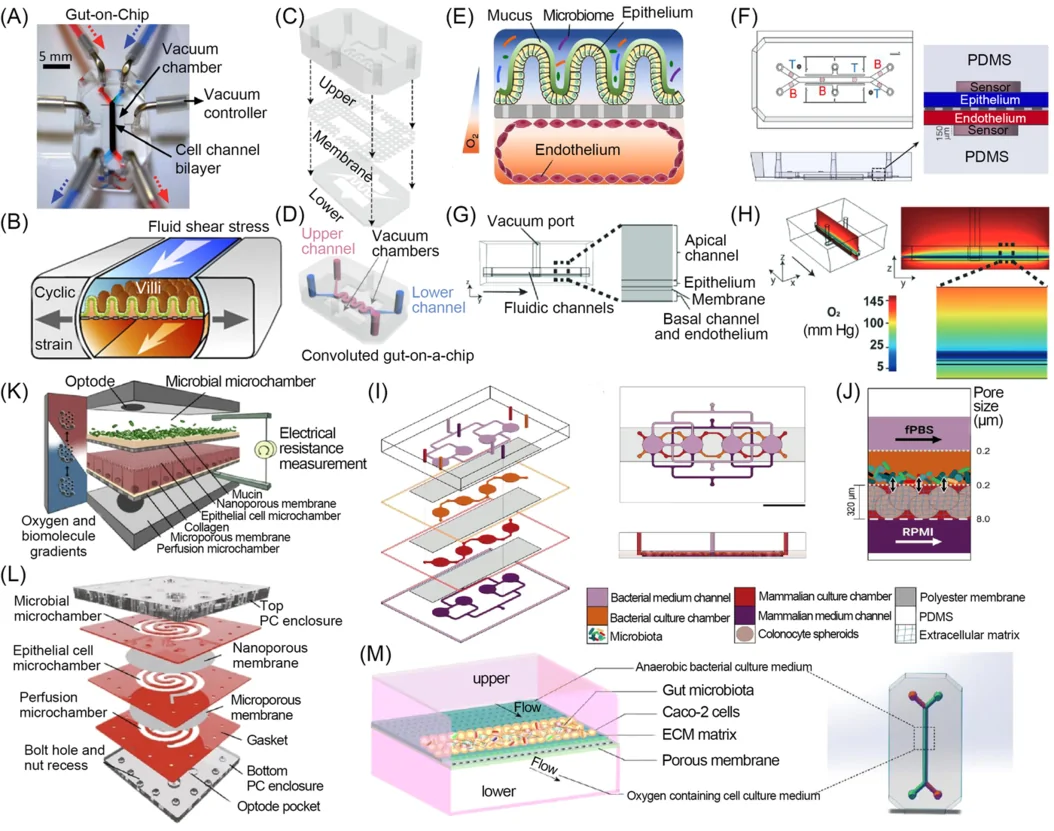
Schematic diagram of the related Gut‐on‐a‐Chip structure. (A) Photographic image of the gut‐on‐a‐chip device composed of clear polydimethylsiloxane (PDMS) elastomer. A syringe pump was used to perfuse (direction indicated by arrows) blue and red dyes through tubing to the upper and lower microchannels, respectively, to visualize these channels. Source: reprinted with permission from ref. [50]. Copyright 2012, with permission from the Royal Society of Chemistry. (B) A schematic of a 3D cross‐section of the device showing how repeated suction to side channels (gray arrows) exerts peristalsis‐like cyclic mechanical strain and fluid flow (white arrows) generates a shear stress in the perpendicular direction. Source: reprinted with permission from ref. [51]. Copyright 2015, with permission from the National Academy of Sciences. (C) and (D) A schematic diagram of the alignment of the upper, membrane, and lower PDMS parts. C: each layer is irreversibly bonded by either plasma or corona treatment. D: a schematic of a fabricated gut‐on‐a‐chip device that has superimposed convoluted microchannels and vacuum chambers. Source: reprinted with permission from ref. [52]. Copyright 2022, with permission from Springer Nature. (E) and (F) Oxygen‐sensitive human Intestine Chip microfluidic culture device. E: the human intestinal epithelium, which is overlaid with its own mucus layer and complex gut microbiota, is positioned over an extracellular matrix‐coated porous membrane (gray). The vascular endothelium lies below the porous membrane. F: a schematic representation of the Intestine Chip with six oxygen‐quenched fluorescent particles embedded in the inlets, middles, and outlets of the top and bottom channels (T and B, respectively). Source: reprinted with permission from ref. [53]. Copyright 2019, with permission from Springer Nature. (G) Side‐view image of the simulated Intestine Chip. The simulated Intestine Chip with the inset showing incorporation of the apical channel, epithelium, membrane, basal channel, and endothelium components in the model geometry. Source: reprinted with permission from ref. [54]. Copyright 2022, with permission from Royal Society of Chemistry. (H) Steady‐state heat map of oxygen distribution in the aerobic Intestine Chip. The oxygen concentration increases along the height of the apical channel away from the epithelium, and the lumen does not sustain a physiologically relevant oxygen level. Source: reprinted with permission from ref. [54]. Copyright 2022, with permission from the Royal Society of Chemistry. (I) and (J) A microfluidic device to study the interaction between bacteria and colonocytes in the colorectal cancer microenvironment. I: exploded, top, and cross‐sectional views highlighting media channels and culture chambers. Scale bar = 1 cm. J: schematic representation of device cocultures. Confined bacterial and colonocyte populations interact via small molecules during perfusion with growth media. Source: reprinted with permission from ref. [55]. Copyright 2024, with permission from the Royal Society of Chemistry. (K) and (L) Conceptual diagram of the HuMiX model for the representative coculture of human epithelial cells with gastrointestinal microbiota (K) and annotated exploded view of the HuMiX device (L). The device consists of a modular stacked assembly of elastomeric gaskets sandwiched between two polycarbonate (PC) enclosures. Each gasket defines a spiral‐shaped microchannel, semi‐permeable membranes affixed to the gaskets separate the channels, with pore sizes chosen for specific functions. A microporous membrane partitions the perfusion and human microchambers, allowing diffusion‐dominant perfusion to human cells. A nanoporous membrane separates the human and microbial microchambers, preventing microorganism infiltration, including viruses, into the human microchamber. Source: reprinted with permission from ref. [56]. Copyright 2016, with permission from Springer Nature. (M) A schematic diagram of the chip channel design and a photograph of the intestine‐chip. Cocultivation of Caco‐2 cells on a chip with specific anaerobic bacteria *Faecalibacterium prausnitzii*. Source: reprinted with permission from ref. [57]. Copyright 2024, with permission from the Royal Society of Chemistry.

In the realm of biomimetic mechanical structure design, researchers have explored the synergistic effects of fluid shear stress and mechanical peristalsis. Studies have demonstrated that fluid shear stress generated by liquid flow within a tube can stimulate tissue oxygen exchange, promote extracellular matrix remodeling, facilitate the formation of basement membranes and microvilli, as well as mucus secretion, thereby improving cytoskeleton and intercellular tight junctions and promoting the formation of the intestinal barrier [48, 49]. Kim et al. [50, 51] developed a three‐channel Polydimethylsiloxane (PDMS) GoC that generates low fluid shear stress (0.02 dyne · cm⁻²) through low flow rates (30 μL/h), while applying cyclic suction to the hollow side chamber to expose epithelial cells to mechanical peristalsis with a 10% deformation frequency. This successfully induced the polarization of small intestinal monolayer columnar epithelium and formed villi structures highly consistent with in vivo conditions (Figure 2A,B). Notably, when mechanical peristalsis was ceased and only tubular flow was maintained, insufficient epithelial deformation led to excessive bacterial growth, exhibiting pathological features highly similar to intestinal obstruction and IBD [51]. This inversely validates the critical impact of missing mechanical stimuli on host‐microbe interactions and provides new insights for pathological model construction. With technological advancements, Shin et al. [52] further achieved the regeneration of functional gut microstructures within 5 days using morphogenesis methods, including three‐dimensional epithelial tissue and crypt‐villus spatial structures, highlighting the trend in mechanical design evolving from two‐dimensional polarization to three‐dimensional complex structures (Figure 2C,D). However, this segment‐adapted mechanical simulation still faces challenges: small intestine chips require precise control of the coupling effect of low fluid shear stress and high‐frequency peristalsis, while large intestine chips need to maintain three‐dimensional structural stability in a stable low fluid shear stress environment, posing higher demands on the fluid dynamics control of microfluidic systems.

Maintaining an anaerobic gradient within the lumen is a prerequisite for host–microbe interaction studies. Research has demonstrated that establishing an intraluminal hypoxia gradient in chips can enhance intestinal barrier function and preserve microbial diversity at physiologically relevant levels [53, 58]. To address this challenge, researchers have developed two strategies: spatial gradients and temporal gradients.

Spatial strategies involve physical separation to achieve zonal control of oxygen concentration. Jalili‐Firoozinezhad et al. [53] employed a nitrogen flushing method to reduce oxygen concentration to <0.5% within 30 min, while diffusing oxygen from the lower endothelial vascular channel through a PDMS membrane to maintain the low oxygen environment required for epithelial cell growth (Figure 2E,F). Grant et al. [54], on the other hand, used an impermeable membrane to regulate oxygen permeability in microchannels, combined with natural oxygen reduction from cellular aerobic respiration to sustain a steady‐state oxygen concentration <6 mmHg within the epithelial lumen (Figure 2G,H). Although both methods can construct physiologically relevant oxygen gradients, their technical logics differ significantly—the nitrogen flushing method relies on an external gas source to rapidly establish an anaerobic environment, suitable for acute experiments; whereas the impermeable membrane method requires no external equipment, offering higher system integration and suitability for long‐term dynamic monitoring.

Temporal strategies involve controlling the sequence of oxygen concentration changes to simulate host‐microbiota symbiosis. Penarete‐Acosta et al. [55] designed a three‐stage model where HCT116 colonic cell spheroids were pre‐cultured at 21% O₂, then transferred to an anaerobic chamber after cell attachment and three‐dimensional proliferation in the chamber. Oxygen levels were reduced to negligible levels within 24 h before introducing microorganisms for coculture, successfully simulating pathogen colonization in colorectal cancer microenvironments (Figure 2I,J). Shah et al. [56] developed the HuMiX chip, integrating oxygen sensors for real‐time dissolved oxygen monitoring. After forming a Caco‐2 epithelial barrier, *Lactobacillus rhamnosus* GG was added, reproducing in vivo transcriptional, metabolic, and immune responses of human intestinal epithelial cells (Figure 2K,L). These technological advancements highlight the evolution from static oxygen control to dynamic temporal regulation. However, maintaining the ecological complexity of microbial communities remains a core challenge—current models often use single or a few strains for coculture, failing to simulate resource competition among indigenous microbiota. Probiotics may also lose their colonization resistance in complex communities.

Notably, GM play a bidirectional regulatory role in drug absorption, distribution, metabolism, and excretion (ADME) processes: microbial enzyme systems can alter drug bioavailability, while drugs like antibiotics can rapidly disturb microbiota structure, leading to the enrichment of antibiotic resistance genes or disruption of gut–brain axis signaling. Nevertheless, current chips only test the functionality of single probiotic strains and do not evaluate their competitiveness with indigenous microbes in environments simulating host communities, potentially resulting in laboratory‐effective strains failing in real gut environments due to colonization resistance loss. Therefore, developing dynamic competition models involving multiple microbial species is a crucial direction to address this issue.

The application of gut‐on‐a‐chip in drug research highlights its technological advantages in replicating biomimetic microenvironments, following a logical progression from molecular mechanism analysis to systemic interaction revelation, and ultimately to intelligent intervention development. Early research focused on the molecular mechanisms of drug action within the gut. The Duodenum Intestine‐Chip (Table 1), targeting the small intestine, successfully predicted the intestinal transport efficiency of oral drugs and drug–drug interactions mediated by CYP3A4 by simulating the polarized cellular structure, gut barrier function, and specific cell subpopulations of the human duodenum [59]. This chip not only reproduced the high expression induction effects of rifampicin and 1,25‐dihydroxyvitamin D3 on CYP3A4 but also quantified the functional activities of drug efflux transporters (MDR1, BCRP, MRP2) and uptake transporters (PepT1, OATP2B1, OCT1, SLC40A1), providing a humanized model for assessing the bioavailability of oral drugs in the gut [59]. As research extended from the single dimension of drug–gut to the multidimensional interactions of gut‐whole body system, GoCs began to delve deeper into the interaction networks between the gut and distant organs during disease progression. Wang et al. [57] developed the Depression‐Gut‐on‐a‐Chip, integrating patient microbiota to successfully reproduce depression‐related pathological features such as reduced intestinal barrier function, chronic low‐grade inflammation, and decreased serotonin levels, offering an in vitro model based on intestinal pathological simulation for studying the mechanisms of psychiatric disorders (Figure 2M). To further enable high‐throughput screening and precise validation of disease intervention strategies, researchers have deeply integrated artificial intelligence technology with intestinal microenvironment regulation. Wu et al. [67] incorporated machine learning into an intelligent GoC, combining the chip with an environmental control system to provide a standardized and scalable intestinal microenvironment for coculturing various probiotics. They successfully evaluated the functional efficacy of 12 *Bifidobacterium* strains in alleviating colitis. This innovative chip + algorithm model signifies the functional transition of GoCs from tools for disease mechanism analysis to platforms for therapeutic target screening. This path of technological iteration driving dimensional expansion not only demonstrates the enhanced scenario adaptability of GoCs in disease research but also provides innovative solutions for precise analysis and efficient treatment of complex diseases through the technological integration of biomimetic design → ecological simulation → intelligent analysis. However, the current simulation of multiorgan system‐level ADME processes by chips is still insufficient and needs to be combined with MOCs such as the gut‐liver axis to improve the evaluation system.

**Table 1:** Representative application evaluation of gut‐on‐a‐chip in disease models or drug screening in the past 5 years.

Organs	Cell populations	Disease	Function	Evaluation	Ref.
Duodenum Intestine	Human duodenal organoid cultures were obtained from biopsies, HIMECs	Drug–drug interactions (DDIs) induced by intestinal drug‐metabolizing enzymes	Showing polarized cell structure, gut barrier function, and specific cell subpopulations; Expressed drug efflux (MDR1, BCRP, MRP2, MRP3) and uptake (PepT1, OATP2B1, OCT1, SLC40A1) transporters; Rifampicin (RIF) and 1,25‐dihidroxyvitamin D3 (VD3) induce high expression of CYP3A4.	Predicting drug transport and CYP3A4‐mediated DDIs of oral medications in the intestine	[59]
Gut	Caco‐2	—	Reduce accidental absorption of small molecules with outstanding anti‐fouling performance and resistance to various biological fluids, small molecule drugs, and plasma proteins, while maintaining cell growth.	Applied to precise drug testing.	[60]
Gastrointestinal tract	Caco‐2, HT29‐MTX‐E12	—	Displaying a complete gut barrier, cells can survive when exposed to a mixture containing chyme.	Used for studying the bioavailability of oral compounds in the fields of pharmacology, toxicology, and nutrition.	[61]
Gut	Caco‐2	—	The presence of mucin layer makes the intestinal epithelial environment more in line with human physiology.	Used to study the adhesion of particles under flowing conditions and the influence of mucosal layer on drug absorption in flowing environments.	[62]
Gut	Caco‐2	—	Barrier‐related proteins are expressed, and gut cell differentiation exhibits spatial distribution, simulating physiological processes related to cell proliferation, lipid transport, and drug metabolism. The permeability of model drugs is highly correlated with human fraction absorbed.	Used to evaluate drug absorption capacity.	[63]
Gut	Caco2, HT‐29, Synbiotics (Engineered from *E. coli* Nissle 1917)	—	Establish a dynamic cortisol sensor with a stable gut environment that senses physiological concentrations of cortisol and activates the gene circuit of tryptophan decarboxylase, converting biologically available tryptophan into serotonin.	Assess the metabolism, production, and transport of tryptophan and tryptamine with human‐relevant apparent permeability. Capable of analyzing cell viability and the secretion of pro‐inflammatory cytokines, and successfully provided an efficacy test for a novel synbiotic.	[64]
Gut	Caco‐2	—	The microfluidic chip model shows lower permeability compared to the transwell model, it can bridge the gap between conventional in vitro and in vivo models.	Evaluate the osmotic efficacy of potent peptide drugs such as insulin and octreotide with gut permeation enhancers (PEs).	[65]
Gut	Caco2‐BBE, HT‐29 MTX, HMVEC, SYNB1618	Phenylketonuria (PKU)	Recapitulated the in vivo activity of synthetic organisms designed for the treatment of PKU.	Used to describe the response of human tissue to engineered bacterial strains and predict the functions of candidate strains in vivo, accelerate the development of synbiotics.	[66]

Abbreviations: Caco‐2, Human colorectal adenocarcinoma cell line; Caco2‐BBE, Human colorectal adenocarcinoma cell line, BBE subtype; HIMECs, Human intestinal microvascular endothelial cells; HMVEC, Human microvascular endothelial cells; HT‐29, Human colorectal adenocarcinoma cell line; HT‐29 MTX, Human colorectal adenocarcinoma cell line, selected for MUC2 expression; HT29‐MTX‐E12, Human colorectal adenocarcinoma cell line, subclone selected for MUC2 expression; SYNB1618, Engineered *Escherichia coli* Nissle 1917 strain for therapeutic purposes.

Despite the significant advancements in biomimetic design, microenvironment simulation, and drug research achieved by GoCs, their technological evolution still faces three major bottlenecks. The limitation of a single functional dimension hinders the accurate simulation of neuro‐gut interaction diseases such as congenital megacolon [68]. Additionally, the adsorption effect of PDMS material on hydrophobic small drug molecules interferes with detection precision [69, 70]. Furthermore, simplified microbiota ecology in single‐strain models fails to reproduce the functional redundancy of complex microbial communities. Future breakthroughs should focus on integrating gut‐nervous‐immune multicellular types to enhance the simulation of complex diseases involving multiple systems. There is also a need to develop surface modification materials, such as coating with perfluoropolyether‐based lubricating layers [60], to mitigate adsorption effects. Moreover, constructing multi‐strain competition‐cooperation models is essential to better reflect the ecological complexity and functional redundancy of indigenous microbiota. From the perspective of technological evolution, GoCs have transitioned from single‐parameter optimization to systemic biomimetic integration. With cross‐disciplinary advancements in materials science, microfluidic technology, and computational modeling, they are poised to become core tools in precision medicine research. Particularly in areas such as microbiome‐host interactions and bidirectional risk assessment of drugs and microbiota, GoCs are expected to unleash even greater potential.

### 3.2 Blood‐brain barrier‐on‐a‐chip

The BBB is a complex system comprising brain microvascular endothelial cells (BMECs) and their tight junctions (TJs), astrocytes (ACs), pericytes (PCs), basement membranes, and extracellular matrix. As the brain's protective barrier, the BBB isolates blood from brain interstitial fluid, restricting molecular transport between the bloodstream and brain parenchyma, thereby shielding the brain from toxins and maintaining neural microenvironment homeostasis [71]. BMECs express specific transporters and receptors that regulate transcellular transport, while TJs between cells limit paracellular diffusion [72]. These features confer remarkable selective permeability to the BBB, only lipophilic small molecules (<500 Da) can cross [73, 74], posing significant challenges for drug development.

Microfluidic technology has introduced a novel paradigm for constructing in vitro BBB models. Conventional BBB‐on‐a‐Chip (BBBoC) designs typically use a porous membrane integrated into the chip to form a bilayer structure, where BMECs and neuroglia are cultured on opposing sides to simulate vascular and neural lumens [75, 76]. Alternative designs employ structures such as microgaps, trapezoids, circles, or multiple repeating paths with shared access ports [77]. BBBoCs generally feature 3D vascular‐like structures and endothelial basement membranes, mimicking cell‐cell interactions in human BBB tissues and physiological properties like fluid shear stress [78].

**Figure 3:**
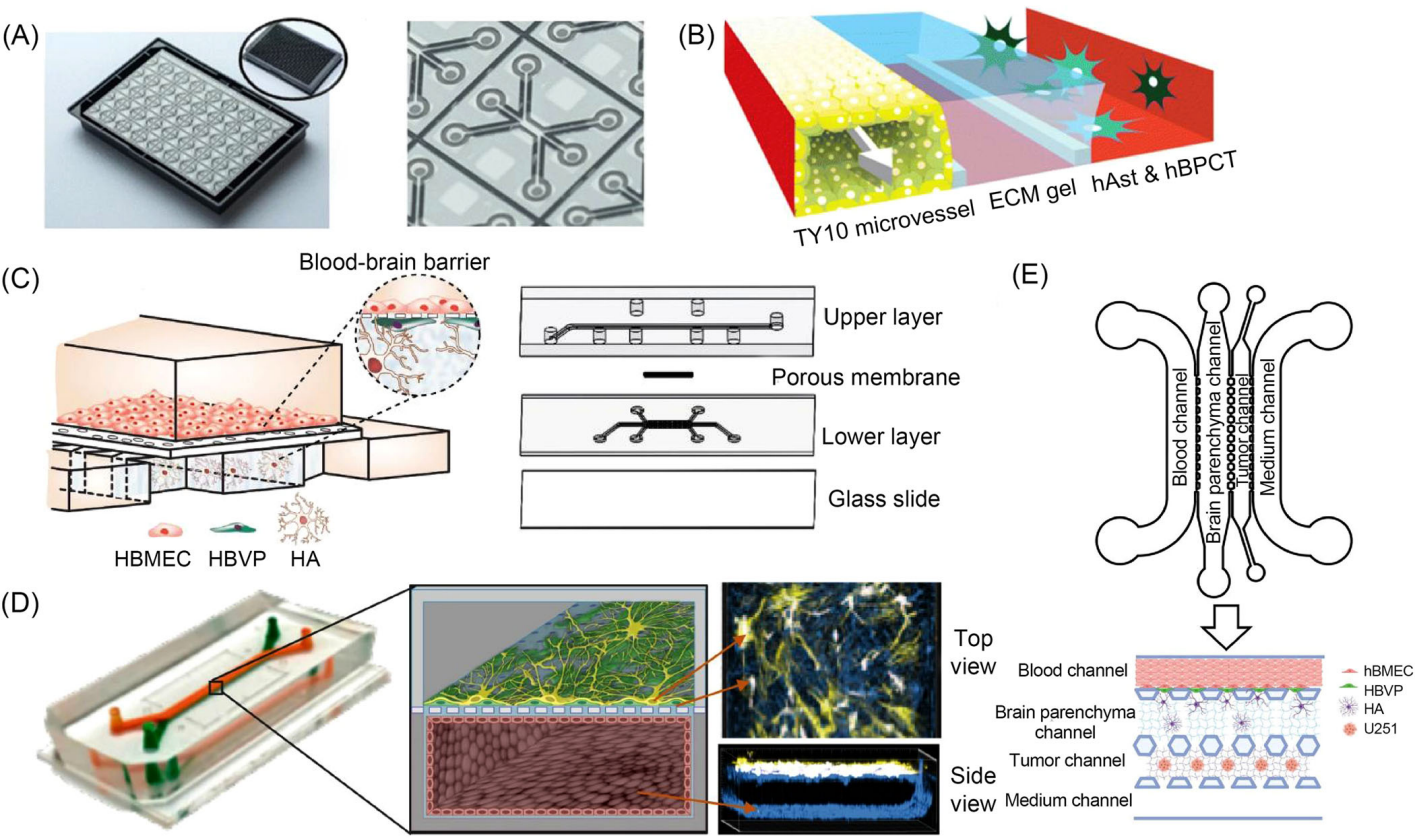
Schematic diagram of the related Blood‐Brain Barrier (BBB)‐on‐a‐Chip structure. (A) The two‐lane OrganoPlate. Left: the plate combines a 384‐well microtiter plate on the top with microfluidic channels on the bottom that make up 96 tissue culture chips. Right: zoom‐in of the bottom of the two‐lane OrganoPlate, showing the tissue culture chips that consist of two channels: a gel channel and a medium channel. Source: reprinted with permission from ref. [79]. Copyright 2018, with permission from Springer Nature. (B) 3D artist impression of the center of a chip. Extracellular matrix (ECM) gel is added to the gel channel, and a phaseguide (phg) prevents it from flowing into the adjacent medium channel. After gelation of the ECM gel, TY10 cells are added to the medium channel, and a TY10 microvessel forms. The microvessel has a lumen at its apical side that is perfused. TY10: human brain microvascular endothelial cell; hAst: human astrocyte; hBPCT: human brain pericyte. Source: reprinted with permission from ref. [79]. Copyright 2018, with permission from Springer Nature. (C) Schematic description of microengineered human BBB model (left) and exploded view of the device consisting of upper vascular layer, porous membrane, lower perivascular layer, and glass slide (right). HBMEC: human brain microvascular endothelial cell; HBVP: human brain vascular pericyte; HA: human astrocyte. Source: reprinted with permission from ref. [77]. Copyright 2020, with permission from Springer Nature. (D) Reconstitution of the human BBB in an Organ Chip microfluidic device. Photograph (left), schematic illustration (middle), and immunofluorescence micrographs (right) of a 2‐channel microfluidic Organ Chip with human induced pluripotent stem‐brain‐like microvascular endothelial cells (BMVECs) cultured on all surfaces of the basal vascular channel, and primary human brain astrocytes and pericytes on the upper surface of the central horizontal membrane in the apical parenchymal channel. At the top right, z‐stack images of the pericytes (yellow, F‐actin staining) and astrocytes (white, GFAP staining) in the top channel of the BBB Chip are reconstituted and shown from above; a side view of similar stacked images for the lower vascular channel containing BMVECs (blue, ZO‐1 staining) is shown at the bottom right. Source: reprinted with permission from ref. [80]. Copyright 2019, with permission from Springer Nature. (E) Schematic illustration of the BBB‐human glioblastoma cell line U251 chip and 3D culture of U251. Source: reprinted with permission from ref. [81]. Copyright 2023, with permission from Elsevier.

Early research on BBBoC primarily focused on biomimetically recreating fundamental barrier functions. Wevers et al. [79] developed a dual‐channel microfluidic platform capable of housing multiple chips, utilizing surface tension techniques for patterned design of extracellular matrix gels to guide BMECs in forming perfusable vessels. They cocultured ACs and PCs on the glial side (Figure 3A,B). This model strictly limited the passage of 20 kDa FITC‐dextran dye and demonstrated significantly higher permeability for antibodies targeting the human transferrin receptor compared to control antibodies (apparent permeability of 2.9 × 10⁻⁵ *vs.* 1.6 × 10⁻⁵ cm/min, respectively), confirming robust barrier function suitable for studying BBB permeability to macromolecules and the differential sensitivity of antibody penetration. It is worth noting that permeability is commonly employed as an evaluation metric for constructing in vitro BBB models. Generally, the apparent permeability coefficient (*P*_app_) of 20 kDa FITC‐dextran in physiological BBB is typically <5 × 10⁻⁶ cm/s. In conventional simplified cellular models, *P*_app_ often exceeds 1 × 10⁻⁵ cm/s. After optimization with cell coculture and fluid shear stress, the *P*_app_ range of models can be reduced to 1 × 10⁻⁶–10⁻⁸ cm/s [82, 83, 84, 85, 86]. However, measured values are often influenced by multiple factors: calibration bias in fluorometer excitation/emission wavelengths may cause fluorescence intensity measurement errors; flow instability in microfluidic systems can directly impact tight junction integrity, inducing deviations in *P*_app_ measurements; variations in cell seeding density or extracellular matrix coating selection may also trigger substantial permeability changes due to differences in barrier maturation. Therefore, strict standardization of experimental conditions is essential to ensure data comparability.

As technology advanced towards integrating multicellular interactions with 3D microenvironments, Ahn et al. [77] designed a chip featuring a 2D endothelial layer combined with a 3D glial network (Figure 3C). This design employed a layered architecture of vascular channel‐porous membrane‐pericyte/astrocyte channel to simulate the vascular‐perivascular niche, providing physiological contact conditions for intercellular signaling. Experience has validated that the model exhibits a *P*_app_ of (1.5 ± 0.2) × 10⁻⁶ cm/s for 4 kDa FITC‐dextran and (0.8 ± 0.1) × 10⁻⁶ cm/s for 40 kDa FITC‐dextran. These values are comparable to BBB substance permeability levels validated in rats in vivo (where *P*_app_ was (0.92 ± 0.46) × 10⁻⁶ cm/s for 4 kDa dextran and (0.19 ± 0.11) × 10⁻⁶ cm/s for 40 kDa dextran, respectively) [87]. It also showed high expression of tight junction proteins ZO‐1 and Occludin, as well as barrier function proteins Glut1 and P‐gp in hBMECs. Additionally, the end‐feet of ACs exhibited specific polarized expression of aquaporin‐4, indicating a high degree of physiological relevance to the barrier. Using fluorescence tracking of HDL‐mimetic nanoparticles with apolipoprotein A1, the chip enabled three‐dimensional mapping of nanoparticle distribution. Combined with scavenger receptor class B type 1 receptor blocking experiments, it confirmed that nanoparticle trans‐BBB transport relies on receptor‐mediated transcytosis, further validating the barrier's functional simulation capability in drug delivery scenarios.

The innovation in cell sources has further expanded the physiological relevance of BBBoC. Park et al. [80] utilized human induced pluripotent stem cells (hiPSCs) to differentiate into iPSC‐BMECs. By simulating embryonic BBB development through a hypoxic microenvironment, they induced high expression levels of tight junction proteins Claudin‐5 and Occludin, as well as the functional transporter P‐gp (Figure 3D). Chip‐integrated electrodes enable real‐time monitoring of transendothelial electrical resistance (TEER), a core electrophysiological metric that quantifies transcellular resistance across endothelial monolayers to assess barrier integrity and functionality [88]. Reported TEER values for BBB chips typically range from 3000 to 20,000 Ω · cm², partially overlapping with the physiological range of healthy human BBB (1500–8000 Ω · cm²) [83, 89, 90, 91]. However, measurement system optimization often introduces deviations: excessive spatial distance between electrodes and the endothelial barrier amplifies resistance contributions from other system components; suboptimal electrode‐to‐cell area ratios disrupt current density uniformity, thereby distorting resistance readings. Additionally, electrode coverage of cells impedes visual monitoring, further compromising measurement reliability. Park's team recorded impedance values of ~25,000 Ω, exceeding prior reports of BBB chip impedance (~400 Ω) with primary hBMECs and perivascular cells by an order of magnitude. This stark increase intuitively demonstrates the barrier‐strengthening effects of hypoxia induction combined with microfluidic coculture. Critically, measurements were not normalized by surface area, yielding results as impedance in Ohms (Ω) rather than resistance (Ω · cm²). Leveraging this high‐fidelity barrier model, the team not only replicated cetuximab's selective BBB transcytosis but also pioneered a novel paradigm of patient‐derived hiPSCs for personalized drug screening, providing a critical technical pathway for precision medicine in neurological disorders.

Integrating disease microenvironments has propelled BBBoCs to the forefront of clinical translation, provides a simulation platform for drug delivery research in various brain diseases (Table 2). Glioma, the most common malignant tumor in the brain, exhibits significant drug resistance due to the interaction between its tumor microenvironment and the BBB [96]. BBB chips address the lack of complex tumor microenvironment models in new drug development for tumors. Shi et al. [81] constructed a BBB‐U251 chip that integrates BMECs, ACs, PCs, and glioma cells (Figure 3E). By monitoring drug permeability across the BBB (apparent permeability coefficients of matrine (1.78 ± 0.20) × 10⁻⁷ cm/s and resveratrol (7.89 ± 3.68) × 10⁻⁸ cm/s) and drug responses in the tumor compartment (matrine inhibited U251 proliferation with a cell survival rate of 84.9 ± 3.5%; resveratrol resulted in a cell survival rate of 74.0 ± 13.3%), they confirmed that the BBB significantly attenuates drug efficacy, underscoring the necessity of BBB‐targeted strategies in anti‐glioma drug development. In conclusion, BBBoC technology has evolved along the path of biomimetic structure construction → physiological function simulation → clinical scenario adaptation, providing a near‐physiological platform for elucidating brain disease mechanisms, drug delivery across the BBB, and precision medicine. However, challenges such as the complexity of multicellular ecosystems, material compatibility, and clinical translation barriers remain critical directions for future technological breakthroughs.

**Table 2:** Representative application evaluation of blood‐brain barrier‐on‐a‐chip in disease models or drug screening in the past 5 years.

Cell populations	Disease	Function	Evaluation	Ref.
iPS‐BECs, PCs, ACs	—	Display stable cell morphology and complete barrier function, capable of distinguishing Blood‐Brain Barrier (BBB) impermeable substances.	Evaluate the transport of drugs through the human BBB.	[60]
hCMEC/D3 s	—	The barrier has selective permeability, and the model can summarize the function of the BBB.	Simultaneous addressing of all eight channels from a common access port can be used for multi‐channel drug testing across the BBB.	[92]
HBMECs, ACs, HBVPs	Brain tumors	Brain tumor cells cocultured in the BBB secrete high concentrations of inflammatory cytokines, exhibiting a more aggressive growth pattern and high drug resistance.	The model can open the BBB chemically to achieve the delivery of non‐permeable drugs, which can be used to study the physiology of the BBB and monitor drug responses based on the interactions between brain tumors and the BBB.	[93]
hCMEC/D3, HPs	AD	Combining sensors to determine cell culture characteristics, with a TEER measurement system close to the barrier.	Used to evaluate the cytotoxicity, permeability, and impact on brain endothelium of targeted gold nanorods in the treatment of Alzheimer's disease.	[94]
hiPSC‐derived endothelial cells, HBVPs, ACs	—	Quantifying the fluorescence intensity inside and outside microvessels can determine the spatial distribution and permeability of fluorescently labeled nanoparticles in the BBB related to human physiological levels.	To evaluate the ability of nanoparticles to cross the human BBB before clinical trials and improve the success rate of clinical trials of neuropharmaceuticals.	[95]

Abbreviations: ACs, Human adipocytes; iPS‐BECs, Human iPSC‐derived brain endothelial cells; hCMEC/D3, Human cerebral microvascular endothelial cell line; HBMECs, Human brain microvascular endothelial cells; HBVPs, Human brain venous endothelial cells; HPs, Human primary pericytes; hiPSC‐derived endothelial cells, Human induced pluripotent stem cell‐derived endothelial cells; PCs, Pericytes.

As therapeutic strategies and drug delivery modalities evolve, BBBoCs have made significant advancements in evaluating novel drug delivery methods such as nanoparticles, adeno‐associated viruses, and stem cell therapies. By integrating three‐dimensional fluid control and multicellular coculture systems, BBBoCs can systematically assess the efficiency of various nanocarriers in crossing the BBB. For instance, doxorubicin‐loaded targeted liposomes can efficiently penetrate the BBB via transferrin receptor‐mediated transcytosis under physiological fluid shear stress [77]. Membrane‐coated nanoparticles utilize the immune cell coculture system within the chip to validate their ability to evade macrophage clearance and prolong their retention time in the brain [97]. In the realm of gene therapy, BBBoCs incorporate reversible barrier regulation techniques and use iPSC‐derived brain endothelial cells to simulate adeno‐associated virus serotype specificity, enabling high‐throughput screening of gene vector delivery pathways [98]. Stem cell therapy research reveals mechanisms by which stem cells regulate BBB function through paracrine pathways. In Parkinson's disease models, it has been demonstrated that barrier integrity directly impacts the ability of mesenchymal stem cell exosomes to repair nigral dopaminergic neurons [99]. These advancements rely on the chip's precise simulation of physiological fluid shear stress, multicellular interactions, and real‐time drug distribution. They not only accelerate the preclinical validation of novel delivery systems, but also drive the evolution of brain disease treatments towards precision, efficiency, and safety.

Despite the significant advancements, BBBoC technology still faces several challenges. Precise control is required over cell ratios, anatomical origins, seeding sequences, medium formulations, and long‐term phenotypic stability in multicellular co‐cultures to achieve optimal conditions for each cell type and maintain model fidelity. Current models often focus on core BBB cells and lack the incorporation of immune cells, neurons, and other distant interactions, which hinders the ability to fully recapitulate the complex pathological networks seen in neurological diseases. The preparation of personalized chips using patient‐derived hiPSCs is time‐consuming, costly, and exhibits poor compatibility with commercial monitoring devices, restricting clinical translation. BBBoCs also struggle to meet the demands for constant flow rates and high‐throughput requirements. Although Xiao et al. [100] developed the constant speed perfusion array chip to address some of these issues, further optimization of chip structure and integrated design is necessary to promote the convenience and commercialization of BBBoCs. Addressing these challenges will be instrumental in enhancing the functionality, reliability, and clinical applicability of BBBoCs, ultimately leading to more effective treatments for brain disorders.

### 3.3 Brain‐on‐a‐chip

The brain, as the most complex neurological organ in the human body, is composed of numerous glial cells and billions of neurons. Neurons are responsible for receiving, integrating, and transmitting information, while glial cells provide support, nutrition, and protection for neurons. Through their rich electrophysiological activities, the brain plays a dominant role in coordinating interactions among various internal organ systems and the external environment [101, 102]. Investigating the unique firing rhythms of neurons and their responses to chemical drugs and external stimuli is crucial for understanding the mechanisms of brain health and disease, and for advancing the development of therapeutic drugs for neurological disorders.

**Figure 4:**
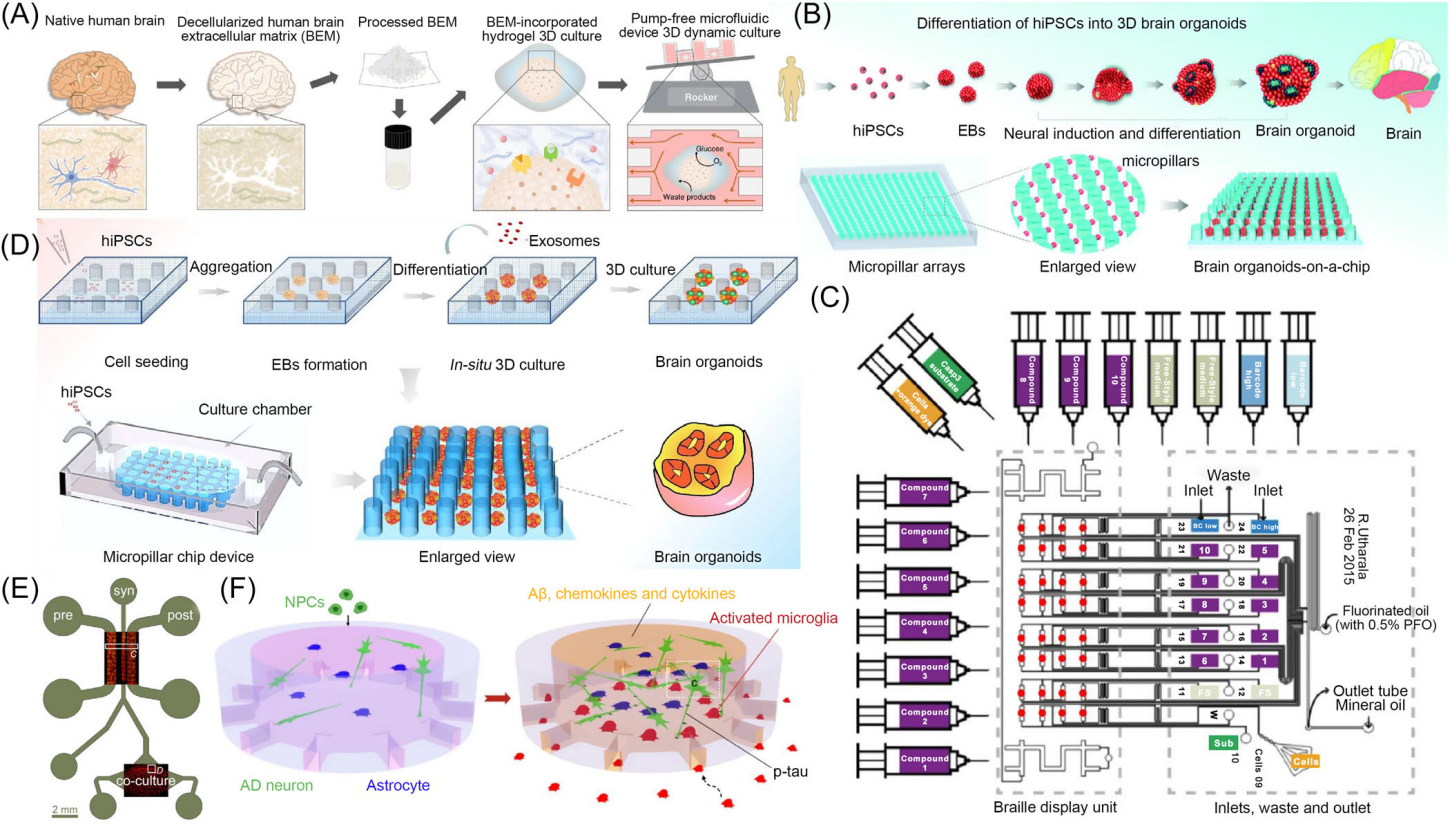
Schematic diagram of the related Brain‐on‐a‐Chip structure. (A) Schematic illustration of the cerebral organoid culture system with a combination of 3D brain extracellular matrix hydrogel culture and the microfluidic device. The cerebral organoid culture system that integrates decellularized brain‐derived extracellular matrix (BEM), extracted and processed from the human brain, into a 3D hydrogel culture system. Utilizing a pump‐free microfluidic device for dynamic 3D culture, the system ensures nutrient supply and metabolic waste removal, effectively replicating the brain tissue microenvironment. Source: reprinted with permission from ref. [103]. Copyright 2021, with permission from Springer Nature. (B) The differentiation of human‐induced pluripotent stem cells (hiPSCs) into 3D brain organoids, involving the formation of embryoid bodies (EBs), neural induction, and differentiation to form brain organoids that ultimately mimic brain structure. Additionally, it showcases the designed “brain organoid‐on‐a‐chip” utilizing micropillar arrays for high‐throughput cultivation of brain organoids. Source: reprinted with permission from ref. [104]. Copyright 2017, with permission from Royal Society of Chemistry. (C) The OrganoPlate® microfluidic platform that integrates multiple injection needles (containing Casps3 substrates, compounds, and cells) with a Braille display unit to automate the processing and screening of compounds with cells. The system utilizes fluorinated oil and mineral oil as fluid media and includes inlets, waste outlets, and sampling outlets for efficient drug screening or cell experiments. Source: reprinted with permission from ref. [105]. Copyright 2018, with permission from Springer Nature. (D) The process of generating brain organoids from hiPSCs on a micropillar chip device, encompassing cell seeding, EBs formation, in situ 3D culture to promote cell differentiation and exosome release, culminating in brain organoid formation, while also displaying the structure of the micropillar chip device and its enlarged view. Source: reprinted with permission from ref. [106]. Copyright 2022, with permission from Springer Nature; (E) The microfluidic chip‐based synaptic model structure, including presynaptic neuron area (pre), synaptic area (syn), and postsynaptic neuron area (post). The presynaptic and postsynaptic regions are connected through the synaptic region to simulate neural signal transmission. The embedded fluorescence microscopy images show the distribution of neurons, and the coculture area at the bottom is used for coculturing other types of cells. The entire structure uses microfluidic channels to isolate different functional regions, enabling precise manipulation and research on synaptic structures. Source: reprinted with permission from ref. [107]. Copyright 2020, with permission from Oxford University Press. (F) Schematics showing multicellular 3D layouts in a microfluidic human Alzheimer's disease culture model. On the left, the normal state includes neural progenitor cells (NPCs), AD neurons, and astrocytes. On the right, the pathological state shows the presence of Aβ, chemokines, and cytokines, leading to the activation of microglia and the secretion of phosphorylated tau protein (p‐tau). Meanwhile, neuronal and astrocytic functions are impaired, simulating the pathological features of AD. Source: reprinted with permission from ref. [108]. Copyright 2018, with permission from Springer Nature.

Microfluidic brain‐on‐a‐chip (BoC) platforms construct in vitro brain function research models through core modules such as neuron and glial cell culture, synapse formation, cell–cell communication, and immune simulation. In the realm of 3D structural innovation, Cho et al. [103] developed a three‐layer, five‐chamber brain‐on‐a‐chip that utilizes a human brain‐mimicking 3D hydrogel matrix combined with boundary element microfluidic technology to achieve bidirectional cerebrospinal fluid perfusion (Figure 4A). This system successfully fosters mature neurons and extensive neural networks during human brain development, spontaneously exhibiting brain morphological features. With the innovation of chamber structures, micropillar arrays have been integrated into devices for 3D cell culture [109]. Zhu et al. [104] introduced an octagonal micropillar array chip that revolutionizes traditional cumbersome processes involving embryoid body formation, neural induction, cell transfer, and 3D encapsulation (Figure 4B). This design allows hiPSCs to undergo embryoid body formation, neural induction, and differentiation in situ, resulting in functional human brain organoids with neuronal differentiation, brain regionalization, and cortical tissue characteristics. It provides an efficient model for studying early neurodevelopmental toxicity, such as cadmium exposure [110]. Both models prioritize precise 3D microenvironment simulation, but Zhu's technology streamlines the differentiation process through in situ integration via micropillar arrays, significantly simplifying operational procedures. This exemplifies how innovative chip structural design can radically enhance the efficiency of organoid culture.

At the level of cell interactions and functional expansion, the critical regulatory roles of non‐neuronal cells, such as glial cells in synapse formation and cell communication, drive the evolution of brain‐on‐a‐chip platforms towards multicellular coculture systems [111]. When iPSC‐derived neurons are mixed with ACs and transferred to the OrganoPlate® platform (Figure 4C), which features a microdroplet plate containing 96 tissue chips, the platform supports 3D culture, coculture, and noninvasive medium regulation. This environment enables rapid formation of neural networks and detection of spontaneous electrical activity, providing a high‐throughput platform for synaptic function research [105]. This design highlights the accelerated value of microfluidic chamber coculture in constructing cellular networks, while the platform characteristics of OrganoPlate® make it uniquely advantageous for high‐throughput screening of neurotoxicity. Beyond fundamental cell interaction studies, Cui et al. [106] combined a micropillar array brain‐on‐a‐chip platform with cortical organoids (Figure 4D) to explore the impact of prenatal environmental exposures on neurodevelopment. This model simulates exposure during pregnancy to breast cancer‐derived exosomes, revealing that treated organoids not only exhibited neuronal damage but also displayed carcinogenic phenomena associated with breast cancer progression. This design not only continues the efficient support of micropillar arrays for 3D organoid structures but also, through in vitro simulation induced by specific pathological factors, offers a high‐throughput research platform for addressing the critical preclinical question of how the tumor microenvironment during pregnancy interferes with neurodevelopment. This exemplifies the innovative application of BoC technology in analyzing environment‐related neurotoxicity mechanisms.

In the context of disease mechanism analysis and drug development, BoC platforms serve as indispensable core modules in MGBA‐MOC research for CNS disorders, demonstrating irreplaceable application value (Table 3). Take Alzheimer's disease (AD) as an example, its pathological features, such as Aβ plaque deposition, Tau protein hyperphosphorylation, and synaptic dysfunction, are closely interrelated [119]. Kilinc et al. [107] developed a three‐chamber microfluidic device that physically separates synapses from pre‐ and post‐synaptic neurons (Figure 4E). By chronically exposing primary hippocampal neurons to toxic Aβ peptides secreted by cells expressing mutant amyloid precursor protein, they observed significant damage to synaptic connections. Further treatment with antibodies targeting different Aβ forms confirmed that low‐molecular‐weight oligomers are key inducers of AD synaptic toxicity. They also discovered that selective expression of protein tyrosine kinase 2β in post‐synaptic neurons can protect neurons from Aβ1–42‐induced synaptic toxicity. Park et al. [108] designed a 3D microfluidic system integrating neurons, ACs, and microglia, which reproduced Aβ aggregation, Tau protein accumulation, microglial recruitment, axonal severing, and NO‐mediated neurotoxicity (Figure 4F). This system provides a multicellular pathological microenvironment simulation platform for studying neuroglial interaction mechanisms and drug discovery. Both types of AD chips focus on in vitro reproduction of pathological features, but Kilinc achieves precise mechanism analysis through physical synapse separation, while Park restores the complexity of pathological processes through tricellular coculture. This reflects the hierarchical application logic of BoC platforms in disease research, ranging from molecular mechanisms to systemic pathology.

**Table 3:** Representative application evaluation of brain‐on‐a‐chip in disease models or drug screening in the past 5 years.

Cell populations	Disease	Function	Evaluation	Ref.
Neurons and ACs	Neuroinflammation	Summarized the pathological features of major neurological diseases: dementia, brain tumors, and brain edema disease‐related microglia can be isolated from heterogeneous populations and allow for selective neuron‐glia involvement in three‐dimensional space.	Explain the interaction between neurons and glial cells in a controlled manner, and verify the association between the activation of innate immune cells and the risk of synaptic damage and neuronal loss.	[112]
Neurons and ACs, BMECs, PCs, *Cryptococcus neoformans*	Fungal brain infection	Summarized the structural and functional characteristics of the blood‐brain barrier (BBB), forming a tightly stable in vitro neurovascular unit with adjacent 3D brain‐like tissue.	Study the mechanism of cross‐cellular mediation and disruption of neuronal homeostasis by pathogens penetrating the BBB, and develop antibacterial drugs for brain infections.	[113]
hiPSC‐derived cortical neurons, hiPSC‐derived BMECs, hiPSC‐derived microglia	Neuroinflammation	Summarized various aspects of human cortical essence and BBB, the model has in vivo and in vitro correlations in identifying crossing differences.	Capture cell‐specific responses in human disease‐related pathology, as well as for screening BBB crossing therapeutic strategies and predicting in vivo responses, promoting translational research and drug discovery.	[114]
Cortical neurons, Striatal neurons	Huntington's disease (HD)	The model reconstructed the cortical striatal network in vitro, which can be used to study presynaptic dynamics, synaptic morphology, and transmission, as well as postsynaptic signal transduction.	The model can be used for drug activity and mechanism of action research, verifying that Pridopidine increases synaptic neurotrophin signaling by rescuing BDNF and TrkB transport in HD treatment, thereby restoring cortical striatal synaptic homeostasis.	[115]
BMECs, ACs, HPs, HMC3, hNPCs, hEPCs, hBMSCs, hAMSCs, hNSCs, hHSCs	Ischemic stroke	The model summarizes the function of the BBB and the interactions between therapeutic stem cells and host cells such as human microvascular endothelial cells, pericytes, astrocytes, microglia, and neurons.	Used to track the infiltration of many candidate stem cells and characterize the expression levels of genes associated with post‐stroke pathology. The model validates that stem cells primarily exhibit unique neurorestorative effects by supporting endogenous recovery, with the restoration of synaptic activity being associated with the recovery of the structural and functional integrity of the neurovascular unit.	[116]
hCMEC/D3, ACs, U87 glioblastoma cells	Brain tumor	The model has formed a complete selective barrier, capable of providing a tumor pathological microenvironment, and shows good performance in antitumor therapy.	The model prevents the diffusion of pectin through the BBB, allowing nanocarriers loaded with chemotherapy drugs to pass through, which can be used for high‐throughput screening of drugs for central nervous system diseases.	[117]
NSCs (murine)	Alzheimer's disease (AD)	It displays brain‐like structures, clear neural differentiation, and neural network formation, and utilizes simple, real‐time, and convenient sensing to monitor the neural network microenvironment.	The AD model can be used for real‐time study of the neurotoxicity of β‐amyloid on neurons and neural networks, thereby enhancing the understanding of neuropathology and accelerating drug discovery.	[118]

Abbreviations: ACs, Human adipocytes; BMECs, Brain microvascular endothelial cells; hiPSC‐derived BMECs, hiPSC‐derived brain microvascular endothelial cells; hAMSCs, Human adipose mesenchymal stem cells; hBMSCs, Human bone marrow mesenchymal stem cells; hCMEC/D3, Human cerebral microvascular endothelial cell line; hEPCs, Human endothelial progenitor cells; hHSCs, Human hematopoietic stem cells; HMC3, Human microglial cell line; hNPCs, Human neural progenitor cells; hNSCs, Human neural stem cells; HPs, Human primary pericytes; NSCs, Neural stem cells; PCs, Pericytes.

Nevertheless, the development of BoC platforms still faces numerous technical challenges. Existing models struggle to accurately simulate the long‐term dynamic pathological processes of brain diseases in vitro: glial cells in coculture are prone to phenotypic drift due to nutrient depletion or microenvironmental imbalances, making it difficult to maintain stable cell ratios over extended periods. Simulating axonal degeneration relies on precise coordination of microfluidic shear forces and chemical gradients, yet current devices lack sufficient capability to control the mechanical‐chemical coupling required for dynamic remodeling of neural synapses. Disease‐related features such as immune cell infiltration gradients and spatiotemporal release of inflammatory factors are even more challenging to fully reproduce in chips. Future advancements need to focus on developing biodegradable material chips that can simulate brain tissue remodeling through controlled material degradation kinetics, thereby extending the functional stability of cells. Additionally, integrating techniques like photothermal neural stimulation and microelectrode arrays can enable real‐time control and monitoring of neuronal activity, aiding in the analysis of neural regulation mechanisms. Furthermore, coculturing patient‐derived hiPSCs with immune cells to construct personalized brain disease models, combined with AI algorithms to predict changes in pathological parameters, can gradually approximate the complex pathological networks of brain diseases. Overcoming these challenges will be crucial for advancing BoC technology, ultimately leading to more effective in vitro models that can better inform our understanding of brain diseases and facilitate the development of targeted treatments.

### 3.4 Multiorgan chip cascade technology

Multiorgan chips (MOCs) involve linking organ‐specific chips via microchannels to study the interactions and connections between various physiological systems and different tissues. Compared to single‐organ chips, MOCs offer greater physiological relevance, better simulating whole‐body systemic responses [120].

**Figure 5:**
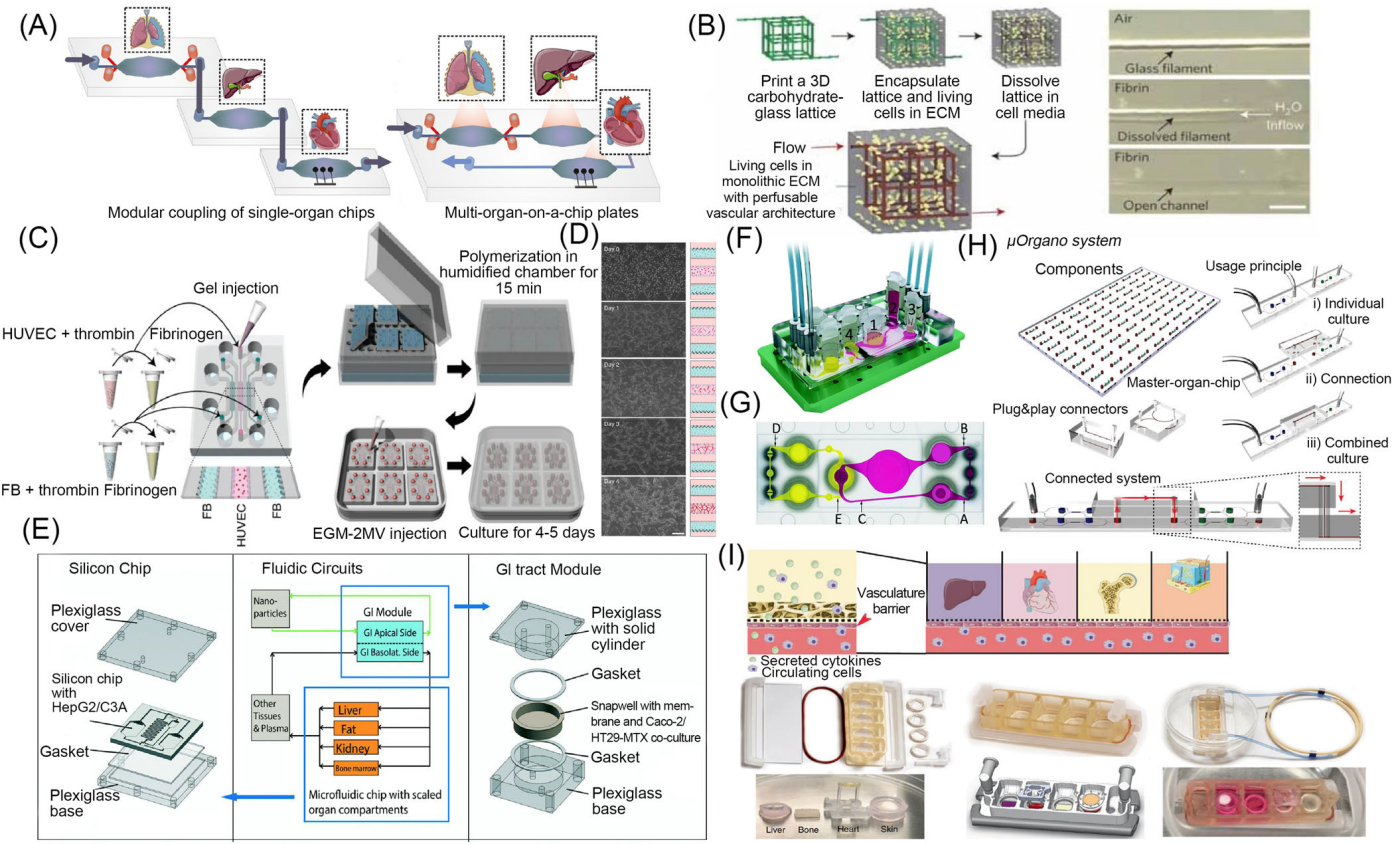
Schematic diagram of the related multiorgan chip structure. (A) Schematic representation of the two main approaches for developing multiorgan chip systems. Source: reprinted with permission from ref. [120]. Copyright 2021, with permission from Elsevier. (B) The process of constructing a perfusable vascular structure using 3D printing technology. Printing a 3D lattice structure composed of carbohydrates and glass, encapsulating living cells within the extracellular matrix (ECM), forming open channels by dissolving the glass lattice in cell culture media, thereby creating a perfusable vascular network within the monolithic ECM, enabling the survival and material exchange of living cells. Source: reprinted with permission from ref. [121]. Copyright 2012, with permission from Springer Nature. (C) Human umbilical vein endothelial cells (HUVECs) and fibroblasts are separately suspended in fibrin gels and injected into parallel gel regions. Upon gel polymerization, media channels are filled with endothelial growth medium and cultured for 4–5d to allow for perfusable lumen formation. Source: reprinted with permission from ref. [122]. Copyright 2017, with permission from Springer Nature. (D) HUVECs elongate, form vacuoles, and subsequently self‐assemble into interconnected perfusable vascular networks over the course of 4–5d. Source: reprinted with permission from ref. [122]. Copyright 2017, with permission from Springer Nature. (E) The design of a multiorgan system module based on a microfluidic chip. The system comprises three parts: the silicon chip, fluidic circuits, and the gastrointestinal (GI) module. The silicon chip includes an acrylic cover, a silicon chip integrated with HepG2/C3A cells, a gasket, and an acrylic base to simulate liver function. The fluidic circuit part connects the GI module with other tissues and plasma, where the GI module is divided into the apical side (Apical Side) and basolateral side (Basolat. Side), and interacts with nanoparticles. The microfluidic chip integrates scaled multiorgan compartments, including functional simulations of the liver, fat, kidney, and bone marrow. The GI module consists of an acrylic solid cylinder, gasket, membrane‐equipped Snapwell, and Caco‐2/HT29‐MTX coculture cells to simulate the gastrointestinal environment. The entire system is fluidically connected to achieve Multiorgan interaction and simulate drug metabolism processes. Source: reprinted with permission from ref. [123]. Copyright 2014, with permission from Royal Society of Chemistry. (F) The Multiorgan chip system structure with four labeled chambers. 3D view of the device comprising two polycarbonate cover‐plates, the PDMS‐glass chip accommodating a surrogate blood flow circuit (pink) and an excretory flow circuit (yellow). Numbers represent the four tissue culture compartments for intestine (1), liver (2), skin (3), and kidney (4) tissue. A central cross‐section of each tissue culture compartment aligned along the interconnecting microchannel is depicted. Source: reprinted with permission from ref. [124]. Copyright 2015, with permission from Royal Society of Chemistry. (G) Evaluation of fluid dynamics in the four‐organ‐chip using micro‐particle image velocimetry. Top view of the four‐organ‐chip layout illustrating the positions of three measuring spots (A, B, and C) in the surrogate blood circuit and two spots (D, E) in the excretory circuit. Source: reprinted with permission from ref. [124]. Copyright 2015, with permission from Royal Society of Chemistry. (H) The design and usage principle of the modular micro‐organ chip system (μOrgano system). The main organ chip contains numerous independent organ units connected via microfluidic channels, supporting high‐throughput organ culture and multiorgan interaction research; plug‐and‐play connectors enable fluid exchange between different chips or organ modules through physical docking, enhancing system flexibility and scalability; the usage principle demonstrates individual culture, connection, and combined culture processes, with organ modules of different colors connected via fluid channels to form an integrated multiorgan system, simulating material exchange and interaction between organs in the body. Source: reprinted with permission from ref. [125]. Copyright 2015, with permission from Public Library of Science. (I) Integrated multiorgan chip enables maintenance of a tissue‐specific niche while allowing for organ cross‐talk. Top: a side view of the multi‐tissue chip where integration is enabled by a vascular barrier beneath each tissue, which creates a tissue‐specific niche in the above chambers for each engineered organ while enabling cross‐talk between organs within the system through the vascular circulation. Bottom: the engineered chip is easily configurable, allowing for a “plug‐and‐play” system for individual organ chambers and a vascular flow channel beneath each organ. Engineered tissues are shown before and after being placed into the engineered tissue chip, where the vascular barrier enables maintenance of each specific medium, as detailed by the differences in medium color within the photograph. Source: reprinted with permission from ref. [126]. Copyright 2022, with permission from Springer Nature.

MOCs are categorized into two types: (1) modular coupling of single‐organ chips and (2) multiorgan‐on‐a‐chip plates (Figure 5A). The modular approach connects individual organ modules using capillary channels or microfluidic motherboards, enabling independent development of single‐organ models and flexible reconfiguration of MOC platforms. This method also supports vascularized organ cultures via organ‐specific endothelial cells [126, 127]. In contrast, multiorgan‐on‐a‐chip plates integrate multiple organs on a single platform, facilitating interorgan communication through microchannels mimicking vasculature. This method provides a paradigm for Human‐on‐a‐Chip, which replicates a miniaturized human circulatory system in a compact design while minimizing leakage risks by eliminating manual interconnections [128].

Chip cascade technology is pivotal for constructing MOCs, with the key challenge being establishing functional vascular networks to deliver nutrients, remove waste, sustain tissue physiology, and enable inter‐organoid communication [129]. Current strategies for in vitro vascularization include 3D printing, vasculogenesis, and angiogenesis [130]. 3D printing constructs large‐scale (>100 µm) vascularized tissues with tunable geometries. Miller et al. [121] created a rigid carbohydrate glass filament network via 3D printing, serving as a cytocompatible soluble template to engineer endothelialized networks mimicking blood perfusion under pulsatile flow (Figure 5B). This method allows independent control of vascular geometry, endothelialization, and extravascular tissue formation, accommodating diverse cell types, matrices, and crosslinking strategies. Sub‐100 µm capillaries can be engineered using endothelial cell emergent behavior via vasculogenesis [131] or angiogenesis [132] strategies. Chen et al. [122] cocultured human umbilical vein endothelial cells and fibroblasts in microchannels, generating microvascular networks via endothelial cell emergent behavior (Figure 5C,D), which recapitulated tumor cell exudation and metastatic behavior.

MOCs can be classified into static, semi‐static, and flexible systems based on cascading modes [133]. Static cascading involves placing specific tissues or organs in individual chambers of a single microfluidic chip, sequentially connected via a common fluid flow. The connection order between the organs is restricted by the permanent geometry of the chip. Esch et al. [123] developed a static MOC containing gastrointestinal, liver, and other tissue modules to study the composite effect of nanoparticle‐tissue interactions (Figure 5E). Its fixed fluidic configuration limits dynamic adjustments. The concept of semi‐static cascade was proposed by Wagner et al. [134]. This approach integrates microfluidic channels, pumps, and sensors to enable pre‐culturing of individual tissues and pre‐configured tissue combinations within one device [135, 136]. Maschmeyer et al. [124] designed a semi‐static four‐organ‐chip with pre‐formed gut and skin models, a 3D liver lobule mimic, and a renal barrier simulated by a monolayer of human proximal tubular epithelial cells (Figure 5F,G). A peristaltic micropump drives pulsatile media flow through interconnected tissue chambers. The flexible cascade offers maximum flexibility, allowing post‐culture integration of organ modules via microtubing or microconnectors [137]. Loskill et al. [125] proposed a modular plug‐and‐play “μOrgano” MOC, where each organ unit comprises a functional master chip and a connective plug‐and‐play connector arranged on an equidistant grid (Figure 5H). They interconnected multiple cardiac chips in series, successfully recapitulating the cellular viability and spontaneous beating rhythms of healthy cardiac tissue. The complexity of MOCs extends further, with the ultimate goal being Body‐on‐a‐Chip systems that replicate whole‐body physiology via Multiorgan cascade technology.

The technological advancement of MOCs provides a robust platform to replicate continuous mediator circulation and inter‐tissue interactions within physiological pathways, enabling comprehensive modeling of the human internal environment for disease pathology, pharmacokinetics, and toxicology studies (Table 4). Ronaldson et al. [126] developed an organ interstitial chip using polysulfone, a biocompatible inert material. This system cultures human‐derived heart, liver, bone, and skin tissues in their respective optimized microenvironments, interconnected via a vascular flow containing immune cells, cytokines, and extracellular vesicles. A selectively permeable endothelial barrier separates tissues from peripheral vascular flow, creating a multiorgan system with interdependent organ functions (Figure 5I). After 4 weeks of culture, interconnected tissues retained normal structural and functional phenotypes and recapitulated the pharmacokinetic and pharmacodynamic profiles of doxorubicin in vivo.

**Table 4:** Representative application evaluation of multiorgan chips in disease models or drug screening in the past 5 years.

Organs	Cell populations	Disease	Function	Evaluation	Primary pathways	Ref.
Gut‐liver axis	Caco‐2, HUVECs, THP‐1, HepaRG, *Escherichia Coli*	colorectal cancer	Support the survival ability and normal function of cocultured gut and liver cells.	Tracking the drug metabolism of colorectal cancer drug irinotecan along the gut‐liver axis; study the complex interactions between gut microbiota and drugs.	Metabolism; Immunity	[138]
Gut‐liver axis	Caco‐2, HT29	—	Simulated the function of the small intestine barrier, enhanced gut permeability and metabolism, and maintained cell viability and phenotype.	Monitor the activation and further metabolism of the prodrug mycophenolate mofetil; Used for drug absorption, distribution, metabolism, and excretion studies to evaluate pharmacokinetic parameters of the combined complex processes of the intestine and liver.	Metabolism; Barrier Function	[139]
Liver, Heart, Lung, Vascular, Colon, Testis, Brain	HSCs, PHH, Kupffer cells, IPSC CMs, hCF, Lonza CC‐2527, MSCs, Lonza CC‐2540S, HUVECs, RCSMCs, Caco‐2, SSCs, Leydig cells, Sertoli cells, HBMECs, HBVPs, ACs, HMs, HOs, HNs	—	The integrated system can maintain the long‐term survival ability of cells and express functional biomarkers.	The multi‐organoid “body‐on‐a‐chip” system can be used to simulate the interdependent metabolism and downstream effects of drugs across multiple tissues in a single platform. It represents a drug screening model that is more in line with physiological characteristics, which can reduce the cost and failure rate of new drug development.	Metabolism; Multiorgan Interactions	[140]
Liver, Gut, Lung, Brain, Heart, Skin, Kidney, BBB	A549 alveolar epithelial cells, HUVECs, PHH, LSECs, Caco2 BBE, A549 human lung carcinoma cell line, Cor4U, Axiogenesis, Human renal proximal tubules, hGMECs, hBMECs, ACs, hNSCs, HDFa, HEK, hDMVECs	—	Eight vascularized dual‐channel organ chips were stably cocultured for 3 weeks, exhibiting good cell viability and organ specificity.	The experimental system, combined with a robotic tracker, quantitatively revealed the metabolic levels and distribution of inulin tracers in various organs. The automated system is expected to reduce drug toxicity, optimize drug administration design, and accelerate the development of medical strategies.	Metabolism; Barrier Function; Multiorgan Interactions	[141]
Liver, Gut, Kidney, Bone marrow	Caco‐2 BBE, HUVECs, PHH, LSECs, Human renal proximal tubules, HGMECs, hCD34, Progenitor cells, HUVECs	—	The channels of the vascular endothelial lining couple the various chip systems in a fluid transfer manner, which supports the long‐term survival ability of cells and simulates the systemic transport of drugs across the endothelial parenchymal tissue barrier.	The pharmacokinetic (PK) parameters of oral nicotine (using gut, liver, and kidney chips) and intravenous cisplatin (using coupled bone marrow, liver, and kidney chips) were predicted. It can serve as a physiological model for human first pass drug absorption, metabolism, and excretion, assist in the conversion of PK and pharmacodynamic parameters from in vitro to in vivo, and is expected to improve the design of drug administration regimens in phase I clinical trials.	Metabolism; Barrier Function; Systemic Transport	[15]
Liver‐Pancreas axis (iPS)	hiPSCs	Type 2 diabetes mellitus (T2DM)	The liver and pancreatic islet tissues in the chip can undergo 3D coculture for up to 30 days under circulating perfusion conditions, and show good growth status and improved tissue‐specific function. Transcriptional analysis reveals the activation of metabolism‐related signaling pathways.	The model promotes insulin secretion in glucose‐sensitive pancreatic islet organoids and increased glucose utilization in liver organoids through glucose tolerance tests. Under high glucose conditions, both the liver and pancreatic islet organoids exhibit mitochondrial dysfunction and reduced glucose transport capacity, and metformin treatment can alleviate these symptoms. The system can reproduce human‐related islet axis under physiological and pathological conditions, and assist in the research and development of drugs for T2DM.	Metabolism; Endocrinology	[86]
Heart, Bone cancer	hiPSCs, MSCs, HTB‐10, RD‐ES‐HTB‐166	Ewing Sarcoma (ES)	Recapitulated the bone microenvironment pathways targeted by linsitinib, and the clinically relevant differences in drug responses between non‐metastatic and metastatic ES tumors.	The integrated engineering of human tumor and cardiac tissue has improved the accuracy of predicting direct and off‐target effects of linsitinib, and this model can be extended to drug response studies in other tissue systems.	Systemic Transport; Drug Response	[142]
Lung‐Brain axis	Vero E6, HPAEpiCs, HULEC‐5a, HBMECs, ACs, HMC3, hPSC‐PCs	SARS‐CoV‐2	The physiological system summarizes the neuropathology associated with SARS‐CoV‐2 infection.	The model can be used to further understand the systemic effects and neurological complications of viral infection, and to demonstrate that systemic inflammation may be the cause of neurological disorders after SARS‐CoV‐2 infection.	Immunity; Neuropathology	[143]

Abbreviations: A549 human lung carcinoma cell line, A549 human lung cancer cell line; A549 alveolar epithelial cells, A549 human lung adenocarcinoma cell line; ACs, Human astrocytes; Axiogenesis, Human cardiomyocyte cell line provided by Axiogenesis company; Caco‐2,; Caco2 BBE, Caco‐2 human colorectal adenocarcinoma cell line, BBE subtype; Cor4U, Cor4U human cardiomyocyte cell line; HBMECs, Human brain microvascular endothelial cells; HBVPs, Human brain venous endothelial cells; HepaRG, Human hepatocellular carcinoma cell line; hCF, Human cardiac fibroblasts; hGMECs, Human glomerular microvascular endothelial cells; HDFa: Human dermal fibroblasts, HDFa subtype; HEK, Human embryonic kidney cell line, HEK293 subtype; hDMVECs, Human dermal microvascular endothelial cells; hCD34, Human CD34+ hematopoietic stem cells; hiPSCs, Human‐induced pluripotent stem cells; HMs, Human microglia; HNs, Human neurons; hNSCs, Human neural stem cells; HTB‐10, HTB‐10 human bladder cancer cell line; HOs, Human oligodendrocytes; HT29, Human colorectal adenocarcinoma cell line; HSCs: Hepatic stellate cells; Human colorectal adenocarcinoma cell line; HUVECs, Human umbilical vein endothelial cells; Human renal proximal tubules, Human renal proximal tubular epithelial cells; HPAEpiCs, Human pulmonary alveolar epithelial cell line; HULEC‐5a, HULEC‐5a human lung microvascular endothelial cell line; HMC3, HMC3 human microglial cell line; hPSC‐PCs, Human pluripotent stem cell‐derived progenitor cells; IPSC CMs, Induced pluripotent stem cell‐derived cardiomyocytes; Lonza CC‐2527/2540S, Normal human bronchial epithelial cells; MSCs, Mesenchymal stem cells; LSECs, Liver sinusoidal endothelial cells; PHH, Primary human hepatocytes; RCSMCs, Rat coronary artery smooth muscle cells; RD‐ES‐HTB‐166, RD‐ES‐HTB‐166 human renal cancer cell line; SSCs, Spermatogonial stem cells; THP‐1, Human monocytic cell line; Vero E6, Vero E6 monkey kidney cell line.

Overall, MOC cascade technology offers a novel strategy for drug development, complementing animal experiments and clinical trials. By mimicking the human body's complex physiological environment under ethical constraints, this technology enables multiorgan‐level drug screening, metabolic profiling, and toxicology assessments. However, MOCs remain largely in the laboratory phase, with commercial applications still underdeveloped, and face several challenges. First, developing physiologically relevant organ and barrier models, along with optimizing environmental perfusion strategies, requires further refinement. Next, due to chip size limitations, cell type composition and ratios must be carefully calibrated during organ scaling to physiologically appropriate sizes [144, 145]. Otherwise, the normal morphology and function of the organs may be affected [146]. Future advancements should focus on enhancing interorgan communication and integrating sensors for multimodal real‐time analysis of organ functional states and biochemical levels under stable coculture conditions.

## 4 MICROBIOTA‐GUT‐BRAIN AXIS‐MULTIORGAN CHIP INTEGRATION AND APPLICATIONS IN DRUG EVALUATION

The intricate etiologies of neurological disorders and the scarcity of effective treatments remain significant challenges in contemporary medicine. The introduction of the MGBA concept offers a promising avenue for addressing these conditions [147]. The development of in vitro integrated OoC platforms based on this axis represents a revolutionary advancement for drug evaluation in neurological diseases. By integrating multiorgan modules and dynamically simulating microenvironments, these platforms have begun to demonstrate substantial potential in drug assessment.

**Figure 6:**
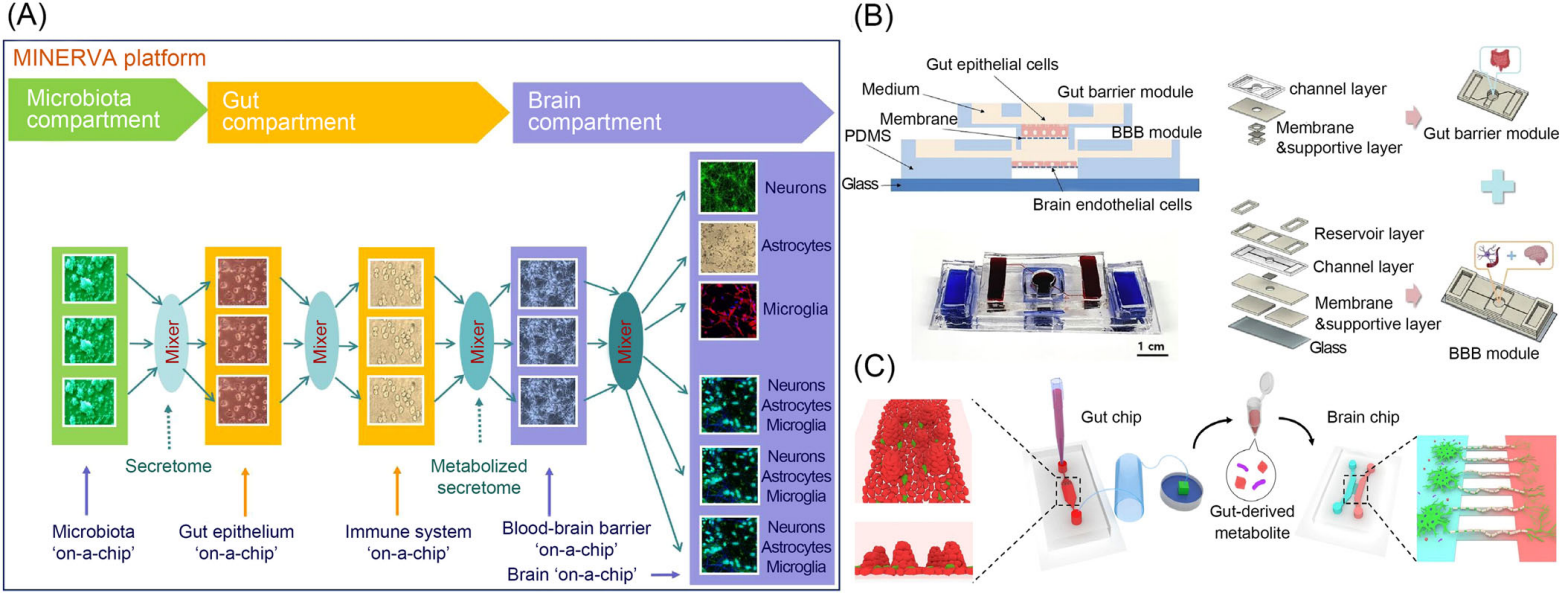
Schematic diagram of the related microbiota‐gut‐brain axis‐multiorgan chips. (A) MIcrobiota‐Gut‐BraiN EngineeRed platform to eVAluate intestinal microflora impact on brain functionality (MINERVA). MINERVA integrates five miniaturized, sensorized, and optically accessible organ‐on‐a‐chip devices, connected in series via microfluidic channels: a 2D culture system for simulating the gut microbiota (green); a gut compartment featuring a 2D culture system for the gut epithelium, coupled with a suspension cell culture system to mimic part of the immune system (yellow); a 2D culture system for modeling the blood‐brain barrier (BBB), and various options for simulating the brain (purple). Source: reprinted with permission from ref. [148]. Copyright 2019, with permission from Elsevier. (B) The design and assembly process of a multiorgan chip system. The system consists of two main modules: the gut barrier module and the BBB module, connected through microfluidic channels. The gut barrier module includes a channel layer, membrane, and supportive layer, with the medium at the top and gut epithelial cells attached to the membrane forming the gut barrier; the BBB module comprises a channel layer, membrane, supportive layer, and reservoir layer, with brain endothelial cells at the bottom forming the BBB. Source: reprinted with permission from ref. [149]. Copyright 2021, with permission from Elsevier. (C) Schematic of the experimental setup for a gut‐brain axis chip system. The gut chip used human epithelial Caco‐2 cells to establish an intestinal lumen, while the brain chip with bridge microchannels guided the axonal growth of neurons. Source: reprinted with permission from ref. [150]. Copyright 2021, with permission from Elsevier.

In 2017, the European Research Council funded the first biomimetic platform project named MINERVA (MIcrobiota‐Gut‐BraiN EngineeRed platform to eVAluate intestinal microflora impact on brain functionality, ID 724734) [147, 148]. This initiative aimed to construct a microbiota‐gut‐brain engineered MOC platform using multiorgan cascade design and hydraulic connections. The platform integrates microbiota on‐a‐chip, gut epithelium on‐a‐chip, immune system on‐a‐chip, BBB on‐a‐chip, and brain on‐a‐chip, organized into three main compartments: microbiota, gut, and brain (Figure 6A). The secretome produced by the microbiota compartment is transmitted to the gut compartment, where it undergoes metabolism and immune processing via the gut epithelium and immune system, resulting in the metabolized secretome. This metabolized secretome then passes through the BBB into the brain compartment, influencing the functions of neurons, astrocytes, and microglia. Connected by mixers, these modules simulate the transfer and interaction of substances across different organ systems, enabling a systematic study of the complex dynamics within the MGBA. Leveraging its capability for multi‐target simultaneous intervention, researchers can assess the effects of drugs on the entire chain of interactions from GM to brain function simultaneously. For instance, when evaluating the therapeutic effects of probiotics on AD, researchers can observe the drug's cascade regulation on GM composition, BBB permeability, and neuroinflammation in the brain, providing a powerful tool for systematic drug efficacy evaluation of microbiota‐drug‐brain interactions.

A mature conceptual model for an integrated MGBA‐on‐a‐chip already exists. Hao et al. [151] invented a biomimetic microfluidic chip (patent number CN202110208528.8), designed to simulate the in vivo signal transmission process of the MGBA. This chip organically integrates GM, intestinal units, the BBB, and brain units to reconstruct the in vivo pathways of microbial metabolism‐immune‐hormone signal transmission. It can be used to study how psychotropic drugs indirectly influence BBB permeability and neurotransmitter levels in the brain by modulating microbiota metabolites, providing a model for analyzing the triangular interaction mechanisms of drug‐microbiota‐brain.

Although comprehensive reports on integrated MGBA‐on‐a‐chip research are not yet available, various teams in the field are actively developing different chip designs and have made some recent advancements. Kim et al. [149] developed a modular Gut‐Brain Axis‐on‐a‐Chip, which uses a glass substrate, PDMS layer, and an intermediate membrane structure to separate the upper and lower layers. The gut barrier and BBB modules are constructed by separately culturing intestinal epithelial cells and brain endothelial cells in upper and lower layers, forming an integrated microphysiological system for studying gut–brain interactions (Figure 6B). Utilizing TEER, the study demonstrated the bidirectional regulation of barrier function by the microbial byproduct sodium butyrate and observed the transport of intestinal exosomes to the BBB, providing an in vitro model for evaluating the brain delivery efficiency of gut‐derived drugs or microbiota metabolites. To further analyze the interaction mechanisms between microbiota and neurodevelopment, Kim's team designed a segmented gut‐brain axis chip model (Figure 6C) [150]. The gut chip simulates the intestinal environment by culturing intestinal epithelial cells (Caco‐2) and GM, producing gut‐derived metabolites. These metabolites are transferred via a microfluidic system to the brain chip, which mimics the brain environment by culturing neural stem cells derived from hiPSCs. The brain chip then studies the effects of these metabolites on neurons and other brain cells. Experiments confirmed that microbial metabolites and exosomes significantly regulate the growth, maturation, and synaptic plasticity of induced neural stem cells, providing evidence for the potential mechanisms of microbiota‐targeted drug interventions in neurodevelopmental or neurodegenerative diseases. Both types of MGBA chip models focus on the controlled simulation of gut‐brain signal transmission, with their ability to characterize barrier function, substance transport, and neurodevelopment across multiple dimensions directly serving neurological drug development. These models can evaluate the ability of gut‐derived drug carriers to cross the BBB, analyze the cascade mechanisms by which microbial metabolites influence neurofunction through changes in intestinal barrier regulation and BBB permeability, and simulate individualized host‐microbiota‐drug interactions using patient‐derived cells and microbiota colonization. This provides technical support for optimizing the delivery efficiency, mechanism analysis, and individualized efficacy prediction of microbiota‐targeted drugs.

Despite the unique advantages of MGBA‐MOC systems in studying gut‐brain interactions, several technical challenges hinder their full integration and application. Including the limitations imposed by the material properties and the difficulty of integrated processing of multiple materials [152, 153], the difficulty in reconciling the distinct microenvironmental and cultural requirements of heterogeneous cell types [154], maintaining long‐term cell viability under dynamic conditions is complicated by external factors like fluid shear stress and metabolite accumulation [155], microscale fluid dynamics complexity [156, 157], balanced control of fluid flow and pressure under multi‐channel conditions [15, 158], real‐time monitoring and multi‐omics analysis of complex signals in chip [159, 160], and functional integration of organ modules—ensuring physiological positioning, connectivity, and activity—requires further optimization [126, 161]. In addition to the above elements, the future MGBA‐MOC integrated system must enhance stability, reproducibility, and scalability to improve reliability and achieve translational application value in biomedical research, particularly for drug development and personalized medicine. In the future, as technological advancements are made in optimizing material biocompatibility, enhancing precise fluid control, and expanding multidimensional detection signals, MGBA‐MOC platforms will gradually transition from mechanism analysis to preclinical drug efficacy evaluation. This progression will provide a near‐clinical research platform for developing microbiota‐targeted drugs for neurological diseases.

## 5 SUMMARY AND OUTLOOK

MGBA research has emerged as a pivotal frontier in gastrointestinal and neuroscientific fields, with model development playing a critical role in elucidating gut‐brain interaction mechanisms. Advances in microfluidic and microfabrication technologies have enabled the creation of dynamic cell culture systems and in vitro models that recapitulate human physiological environments. Current MGBA models leveraging these technologies are already advancing foundational research, demonstrating exceptional utility in drug toxicity prediction and neuropathological simulation. By refining preclinical studies of drug ADME and enhancing toxicity assessment accuracy, such platforms promise to reduce drug development costs, improve clinical trial success rates, and drive transformative advancements in personalized medicine and healthcare.

As a cutting‐edge platform for studying complex organ interactions and disease mechanisms, MGBA‐MOCs demonstrate immense potential in disease modeling and drug testing but face significant technical hurdles for their advancement. Future MGBA‐MOCs must focus on precisely replicating the gut‐brain microenvironment, and integrating patient‐derived organoids or stem cell‐engineered tissues into MGBA‐MOCs represents a promising strategy to enhance physiological relevance and predictive accuracy. Concurrently, embedding advanced biosensors and real‐time monitoring technologies will be critical to analyze dynamic cellular and molecular responses within the chip.

The evolution of MGBA‐MOCs will not only deepen insights into gut‐brain crosstalk but also offer novel perspectives for dissecting complex diseases like IBD and neurodegenerative disorders. To realize this vision, the field demands a multidisciplinary convergence of expertise in microfluidics, tissue engineering, biosensing, and additive manufacturing—overcoming current bottlenecks to unlock the platform's full potential in revolutionizing drug discovery and personalized medicine.

## AUTHOR CONTRIBUTIONS

Yue Tang and Hewen Chen surveyed the literature and drafted the article. Yue Tang, Ziyue Zhao, Xuesong Kang, Wenxin Wang, Kun Dai, and Yufei Guo wrote the manuscript with input from all co‐authors. Zikai Hao, Axin Liang, and Aiqin Luo edited the manuscript and supervised the work. All authors read and approved the final manuscript.

## CONFLICT OF INTEREST STATEMENT

The authors declare no conflicts of interest.

## ETHICS STATEMENT

This article does not contain any studies with human participants or animals performed by any of the authors.

## Data Availability

No new data were generated or analyzed in this review. Supplementary materials (graphical abstract, slides, videos, Chinese translated version, and update materials) may be found in the online DOI or iMeta Science http://www.imeta.science/imetaomics/.
